# Changes in reproduction mediate the effects of climate change and grassland management on plant population dynamics

**DOI:** 10.1002/eap.3063

**Published:** 2024-12-08

**Authors:** Martin Andrzejak, Tiffany M. Knight, Carolin Plos, Lotte Korell

**Affiliations:** ^1^ Ecology and Genetics University of Oulu Oulu Finland; ^2^ National Tropical Botanical Garden Kalāheo Hawaii USA; ^3^ Department of Species Interaction Ecology Helmholtz Centre for Environmental Research GmbH–UFZ Leipzig Germany; ^4^ Institute of Biology, Geobotany and Botanical Garden, Martin Luther University Halle‐Wittenberg Halle Germany; ^5^ German Centre for Integrative Biodiversity Research (iDiv) Halle‐Jena‐Leipzig Leipzig Germany

**Keywords:** climate change, global change experimental facility, grassland management, integral projection model, life table response experiment, mowing, plant population dynamics, sensitivity analysis, sheep grazing

## Abstract

Climate change is one of the largest threats to grassland plant species, which can be modified by land management. Although climate change and land management are expected to separately and interactively influence plant demography, this has been rarely considered in climate change experiments. We used a large‐scale experiment in central Germany to quantify the effects of grassland management, climate change, and their joint effect on the demography and population growth rate of 11 plant species all native to this temperate grassland ecosystem. We parameterized integral projection models with five years of demographic data to project population growth rate. We hypothesized that plant populations perform better in the ambient than in the future climate treatment that creates hotter and drier summer conditions. Further, we hypothesized that plant performance interactively responds to climate and land management in a species‐specific manner based on the drought, mowing, and grazing tolerances as well as the flowering phenology of each species. Due to extreme drought events, over half of our study species went quasi extinct, which highlights how extreme climate events can influence long‐term experimental results. We found no consistent support for our expectation that plants perform better in ambient compared with future climate conditions. However, several species showed interactive responses to the treatments, indicating that optimal management strategies for plant performance are expected to shift with climate change. Changes in population growth rates of these species across treatments were mostly due to changes in plant reproduction. Experiments combined with measuring plant demographic responses provide a way to isolate the effects of different drivers on the long‐term persistence of species and to identify the demographic vital rates that are critical to manage in the future. Our study suggests that it will become increasingly difficult to maintain species with preferences for moister soil conditions, and that climate and land use can interactively alter demographic responses of the remaining grassland species.

## INTRODUCTION

Grasslands cover approximately 40% of the Earth's surface (Bardgett et al., 2021) and provide important ecosystem services to society, such as food for livestock and humanity, pollination, and biodiversity (Biurrun et al., 2021; Feurdean et al., 2018). Seminatural grasslands in central Europe have been historically created and maintained by traditional agricultural practices that prevent their succession into forests (Poschold et al., 2009). Most commonly, the management of such seminatural grasslands is low‐intensity (i.e., extensive) mowing or grazing (Tälle et al., 2016). These different types of management affect which types of plants can persist, as species differ in their ability to tolerate grazing and mowing (Briemle et al., 2002; Busch et al., 2019). Anthropogenic climate change may modify the effects that management has on the demography and persistence of plant species (Bardgett et al., 2021; Kariyeva et al., 2012; Redlich et al., 2022). For example, individuals that currently avoid loss of reproduction from mowing by flowering before or after mowing events might shift in their phenology in the future due to climate change, which could lead to mismatches between flowering timing and mowing events that harm plant reproductive success (Cleland et al., 2006; Gordo & Sanz, 2010).

In central Europe, the climate is projected to become warmer, wetter in spring and fall, and drier in summer in the future (Döscher et al., 2002; Jacob & Podzun, 1997; Rockel et al., 2008; Wagner et al., 2013). Drought‐tolerant species should be best able to survive the drier summers expected in the future (Belovsky & Slade, 2021). Species that flower earlier and across a longer time period might be favored by future climate conditions because they can take advantage of the wet spring conditions. Furthermore, the long flowering duration might buffer their populations from complete reproductive failure during periods of extreme weather. This leads to the expectation that drought‐tolerant plant species with an early flowering start combined with a long flowering period should be best able to tolerate future climate conditions.

The effects of land use on plant populations may depend on species traits (e.g., phenology) that shape their tolerances against mowing and grazing. Species with an earlier flowering start should be favored by the mowing treatment because many can reproduce before the mowing event and because grazers foraging early in the season may consume their flowers and fruits before they can ripen to seeds (Wentao et al., 2023). Because many traits are involved in grazing and mowing tolerances, plant indicator values that are based on expert knowledge on the species tolerances (Klotz et al., 2002) can help to explain the response of plant species to different land management types (Briemle et al., 2002; Busch et al., 2019).

In order to understand the population dynamics of plant species, demographic studies are used to quantify vital rates such as survival, growth, and reproductive output across the entire life cycle of the plant and project the long‐term population growth rate using structured population models (Caswell, 2000; Crone et al., 2011; Easterling et al., 2000). A common approach for analyzing plant demographic data is the use of integral projection models (IPMs), which consider the relationship between continuous plant size and vital rates and can also incorporate discrete development stages (Childs et al., 2003; Jacquemyn et al., 2010; Yule et al., 2013). Retrospective analyses of IPMs, such as life table response experiments (LTREs), decompose the contribution of individual vital rates to the changes in the population growth rate between treatments. Vital rates that are responsible for the change are those that either change dramatically between the treatments and/or those that the population growth rate is sensitive to changes in (Rees & Ellner, 2009).

Many demographic studies have been conducted to assess how the demography of species responds to climate (Ehrlen, 2019; Ehrlén et al., 2016; Ehrlén & Morris, 2015; Lemmer et al., 2021; Morris et al., 2020). Most of these studies focus on natural variation across space and time in climate conditions and measure plant demographic responses (Compagnoni et al., 2021). Such observational studies are limited in their ability to assess cause and effect relationships, as there are often other important factors that covary with climate. A few studies have experimentally assessed how climate affects plant demography (Compagnoni & Adler, 2014; Lemmer et al., 2021; Lyu & Alexander, 2022; Töpper et al., 2018; Williams et al., 2007). However, most climate change experiments consider extreme manipulations of temperature and precipitation, making it hard to predict how plants might respond under realistic future climate change scenarios (Korell et al., 2020).

We have a unique opportunity to study how a realistic future climate scenario as well as two different grassland management types (extensive sheep grazing vs. mowing) influence the demography and population dynamics of plants using the Global Change Experimental Facility (GCEF) in Germany. Our study builds on the research of Lemmer and colleagues (Lemmer et al., 2021), who discovered that land management types and climate change interactively affected the population growth rate of the drought‐tolerant grass, *Bromus erectus* L., and that this interaction was primarily due to higher seedling recruitment of plants under the future climate and mowing management treatment combination. Lemmer et al. (2021) measured the demography across a short time period (one transition, two years) and concentrated on a single species. In this study, we expand the scope to understand the demography and population responses to climate and land management types across multiple transitions along a longer time period and multiple plant species. Demographic data were collected over the course of five years (2018–2022) for 11 (Table 1) grassland plant species that represent a range of different plant statures (tall vs. small), life forms (grasses, herbs), and life histories (biennials vs. perennials). In general, we expected (1) the effects of land management types will vary across species and depend on indicator values for grazing and mowing tolerance, (2) the future climate treatment will decrease plant population growth rates, and (3) climate treatments and land management types will interactively influence population growth rates for each species.

**TABLE 1:** Description and ecology of each plant species in the study as extracted from the following sources: ^a^ https://wiki.ufz.de/biolflor/index.jsp and ^b^Ellenberg et al. (1991).

Species name	Growth form	Plant family	Life history^a^	Quasi extinct	Ellenberg^b^	Start of flowering	End of flowering	Duration of flowering	Unit of measure	Grazing resistance^a^	Mowing tolerance^a^
*Anthoxanthum odoratum* L.	Grass	Poaceae	p^1^	†	Ind^4^	April	April	1	Basal	5^10^	7^15^
*Bromus erectus* Huds.	Grass	Poaceae	p	*	3^5^	May	June	2	Basal	4^11^	5^16^
*Crepis biennis* L.	Forb	Asterace	b^2^	†	6^6^	June	August	3	No.	2^12^	6^17^
*Dianthus carthusianorum* L.	Forb	Caryoph	p	*	3	April	December	9	Basal	4	3^18^
*Lotus corniculatus* L.	Legume	Fabacea	p	†	4^7^	June	July	2	Basal	4	6
*Lychnis flos‐cuculi* L.	Forb	Caryoph	p	†	7^8^	NA	NA	NA	Basal	2	4^19^
*Medicago falcata* (L.) Arcang	Legume	Fabacea	p	†	3	June	July	2	Basal	2	5
*Plantago lanceolata* L	Forb	Plantagi	p	*	Ind	May	September	5	No.	6^13^	7
*Scabiosa ochroleuca* L.	Forb	Dipsacac	p	*	3	April	December	9	Basal	NA^14^	NA
*Tragopogon orientalis* L.	Forb	Asterace	h^3^	*	5^9^	May	December	8	No.	NA	NA
*Trifolium pratense* L.	Legume	Fabacea	p	†	5	May	May	1	Basal	4	7

*Note*: The flowering duration (monthly start and end of flowering) for our surviving species was measured in the field in 2020. In the column quasi extinct † indicates species that went extinct and * indicates species that were included in this study. ^1^Perennial and iteroparous, ^2^biennial, ^3^pluriennial‐hapaxanthic, ^4^Indifferent, ^5^species is more common at dry sites, avoids wet habitats, ^6^prefers semi wet to wet locations, ^7^usually more on dry habitats, able to grow on wet sites, ^8^prefers wet locations, without waterlogging, ^9^prefers intermediate locations, ^10^moderately tolerant to grazing, ^11^sensitive/moderately tolerant to grazing, ^12^intolerant/sensitive to grazing, ^13^moderately tolerant/well tolerant to grazing, ^14^no information available, ^15^well tolerant to mowing, ^16^moderately tolerant to mowing, ^17^moderately to well tolerant to mowing, ^18^sensitive to mowing, ^19^sensitive to moderately tolerant to mowing.

## METHODS

### Study site

We conducted our research in the GCEF, which was established in 2013. The experiment is part of the research station Bad Lauchstädt (51°22060 N, 11°50060 E, 118 m above sea level). Mean annual precipitation is 489 mm and mean annual temperature is 8.9°C (1896–2013, Schädler et al., 2019). The GCEF is designed to simulate a realistic future climate scenario combined with the manipulation of different land management types, including extensively managed grasslands. The future climate treatment is based on a mean of 12 regional climate models for the region (Schädler et al., 2019).

The GCEF consists of 10 main units, five with ambient climate and five with future climate conditions. Precipitation in the future climate treatment is manipulated seasonally: increases in precipitation by +10% compared with the ambient climate in spring and fall, and reduced precipitation by −20% compared with the ambient climate in summer (for more details see Schädler et al., 2019). The minimum temperature in the future climate treatment is increased by about +1.5°C by passive nighttime warming (Schädler et al., 2019).

Each main unit is divided into five subunits that are 16 × 24 m in size, each representing a different land management. Each subunit has 14 × 19.5 m available for measurements and the remainder is left as an edge area, where no experiments or measurements can be undertaken (Appendix S1: Figure S1). Our study was established in two of the five land management types, the extensive meadow (mown one or two times per year) and the extensive pasture (grazed by 20 sheep for 24 h, 2–3 times per year). These two land management types were initially sown in 2013 with 56 plant species native to Germany (Schädler et al., 2019). This design leads to the following treatment combinations in our experiment: (1) ambient grazing, (2) ambient mowing, (3) future grazing, and (4) future mowing.

### Study species

In 2018, we chose 11 different species (Table 1) for our demographic study based on the criteria that the species were abundant enough for demographic data collection (minimum number of individuals in 2018 was 50 per treatment combination), and that they are typical for mesic or dry grasslands. The species cover a range of growth forms, plant families, and life histories (biennial vs. perennial). Every plant species in this study is native to Germany and was sown into the GCEF experiment when it was established.

### Demographic data collection

In April 2018, we established one transect on all our study subunits resulting in 20 transects (5 replicates per treatment combination). Our goal was to find a minimum of 10 individuals per transect to reach a minimum of 50 individuals per treatment combination for every species. Along each transect, 50 × 50 cm subplots were established. We assigned three of the subplots to fixed positions along each transect (0–0.50 m; 3–3.50 m; 6–6.50 m). The location of other subplots was chosen at strategic locations along the transect to capture individuals of species that were needed to achieve our target of 50 individuals per species and treatment combination. Following this procedure, most transects had eight subplots. This leads to a different overall subplot number for each species, with rarer species having more subplots than common species. Each subplot was marked with a land marking (pipe with red plastic top) in the bottom left and a nail with a white plastic ring in the top right corner.

In April 2018, we measured the size of each individual target species in each subplot using either basal area or number of leaves (Table 1). Basal area (in square centimeters) was measured as the product of the horizontal width and length at the base of each individual target species. Length was measured as the longest lateral dimension of each plant, and width was measured perpendicular to the length. For rosette species (e.g., *Plantago lanceolata*, *Tragopogon orientalis*), we used the number of leaves as proxy for their size. We recorded the location of each individual within the 50 × 50 cm subplot, using an *XY*‐coordinate system so that individuals could be followed over the years. In rare cases, if we spotted individuals of our rarer species just outside of our subplot, we would measure those individuals and track their position relative to the subplot (one of the coordinates would exceed 50 cm).

From 2019 until 2022, we went to the field five times a year. In April, we relocated individuals from the previous year and measured their size, identified new individuals and measured their size, and counted the number of new seedlings (for details on how we identified seedlings see Appendix S1: Table S2). Depending on the phenology of the species and the management events (see Table 1; Appendix S1: Table S1), we counted flowers and seed heads of all reproductive individuals in each transect. We collected two seed heads per subunit and species from individuals that were outside of our transects but within the same subunits. Number of seeds in each seed head were counted in the lab. In late autumn (October/November), we counted the number of seedlings in our subplots, as seedlings of these species can germinate in spring or in fall.

From 2019 onward, we decided to mark individuals of the very abundant species *B. erectus* with IDs as that helped separating and finding individuals. To mark the individuals, we used rings made of wire with a tag with a running number on it and put it around the base of the individual. We left enough space for the individual to grow. Special care was taken to not harm any of the vegetation during that process.

The multiple droughts Germany experienced during several of the years of this study prevented more frequent mowing and grazing than would otherwise be incorporated into the treatment plan (Schädler et al., 2019). During the time of our experiment, the extensive meadows were mown once per year in early summer time from 2018 to 2020 and two times (early and late summer) in 2021. The extensively grazed subplots were grazed twice per year in spring and early summer from 2018 to 2020 and three times in 2021 (spring, early and late summer). For a detailed look at the management timing, see Appendix S1: Table S1.

Given the large number of potential traits involved (e.g., hairiness, regeneration capacity) in species abilities to tolerate grazing and mowing, species indicator values that are based on the relative performance of species in response to grazing or mowing based on expert knowledge (Briemle et al., 2002) are useful proxies (see e.g., Busch et al., 2019). We used Ellenberg indicator values for drought tolerances (Ellenberg et al., 1991), grassland utilization indicator values for grazing and mowing tolerances (Briemle et al., 2002) from the Bioflor database (Klotz et al., 2002), and our own measurements of phenology to explain differences in the response of species to the management and climate treatments (Table 1). From April through December 2020, we collected monthly phenology data to establish the start and end of flowering for each target species in the GCEF within the central 3 × 3 m vegetation survey plot of each subunit where no experiments or other disturbances (besides the management events) occur (Schädler et al., 2019).

### Quasi extinction

Some of the species declined so significantly over the course of our demographic study that we considered them quasi extinct and no longer continued to collect demographic data. We defined quasi extinction from a treatment combination as fewer than 25 individuals total and fewer than 10 flowering individuals.

### Life cycle stages and vital rates

**FIGURE 1:**
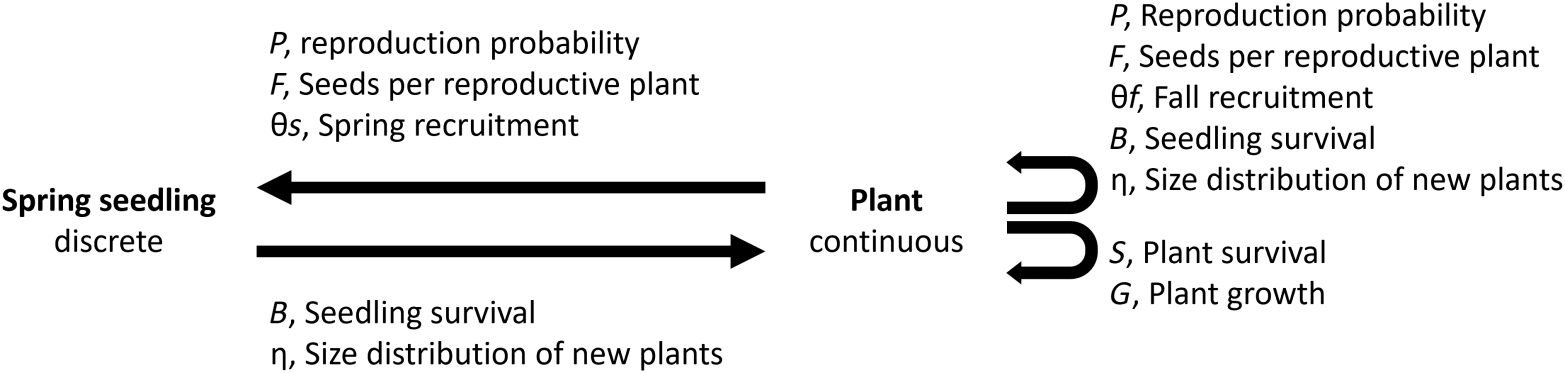
Life cycle diagram of the study species. The diagram shows every vital rate incorporated in the integral projection model and the abbreviation used.

For each species, we modeled a year‐to‐year life cycle (April to April) with two stage classes. A discrete class for seedlings and a continuous class for plants (Figure 1). Most of our species do not form long‐lasting seed banks, and thus we do not have a seed bank class. *P. lanceolata* does have a long‐lived seed bank (Chen, 2018), but most of the seeds of this species germinate immediately and, therefore, demographic studies on this species do not typically incorporate the seed bank stage (Buckley, 2023).

We modeled each continuous state of the IPM as a function of the natural logarithm of the size of an individual because size is a good predictor of several vital rates (survival, growth, reproduction probability, and number of seeds) for all species (Appendix S1: Table S3, Figures S10–S13). Survival is a function of whether or not an individual (*i*) was still alive at *t*1, dependent on its log transformed size (*z*) at *t*0 (Equation 1), modeled as a Bernoulli process with probability of survival *S*_*t*1_ (Equation 2).

(1) $$S_{i,t1}\sim \text{Bernoulli}\;\left({\hat{S}}_{t1}\right).$$

(2) $$\text{logit}\left({\hat{S}}_{t1}\right)=\alpha +\beta \;\log_{e}\left(z\right).$$

In this function, α is the intercept and β the slope of the curve.

Growth of plants *G*_*i*,*t*1_ is described as the normally distributed change in *z* and log transformed size at *t*1 (*z*′) of a surviving individual plant (*i*) (Equation 3). It was modeled as a linear function of *z*′ of surviving individuals depending on *z*, where the intercept is defined as $\alpha^{G}$, the slope as $\beta^{G}$ (Equation 4), and the SD as σ_*G*_ (Equation 3).

(3) $$G_{i,t1}\sim \text{Normal}\;\left({\hat{G}}_{t1},\sigma_{G}\right).$$

(4) $${\hat{G}}_{i,t1}=\alpha^{G}+\beta^{G}\;\log_{e}\left(z\right).$$

Similar to survival, we modeled reproduction probability (*P*_*i*,*t*0_) as a Bernoulli process (Equation 5). Here, we tested how the probability an individual *i* reproduces at time *t*0 depending on *z*. The intercept is defined as $\alpha_{t}^{P}$ and the slope as β^*P*^ using a logit link function (Equation 6).

(5) $$P_{i,t0}\sim \text{Bernoulli}\;\left({\hat{P}}_{t}\right).$$

(6) $$\text{logit}\left({\hat{P}}_{t0}\right)=\alpha_{t}^{P}+\beta^{P}\;\log_{e}\left(z\right).$$

For every reproductive plant, we calculated the number of seeds it produced (Equation 7). The number of seeds produced by an individual *i* at *t*0 was a function of *z*, modeled as a Poisson distribution. The intercept was defined as $\alpha_{t}^{F}$ and slope β^*F*^ (Equation 8).

(7) $$F_{i,t0}\sim \text{Poisson}\left({\hat{F}}_{t0}\right).$$

(8) $${\hat{F}}_{t}=\alpha_{t}^{F}+\beta^{F}\;\log_{e}\left(z\right).$$

Recruitment, the emerged seedlings per total number of seeds, was separated into fall (θ_*f*,*j*,*t*0_) and spring recruitment (θ_*s*,*j*,*t*0_). To calculate recruitment, we summed the total number of seeds per subplot (*S*_*j,t*_, Equation 9), where *S*_*i,t*1_ is the total number of seeds produced by one individual (*n*) in a subplot at time *t*1.

(9) $$S_{j,t}=\sum_{1}^{n}S_{i,t1}.$$

We then divided the result of Equation (9) by the number of seedlings found in that subplot in fall *t*0 (*Rf*_*j,t0*_, Equation 10) and spring *t*1 (*Rs*_*j*,*t*1_, Equation 11).

(10) $$\theta_{f,j,t}=\hat{R}f_{j,t0}/S_{j,t}.$$

(11) $$\theta_{s,j,t+1}=\hat{R}s_{j,t1}/S_{j,t}.$$

Establishment (*B*_*j*_) is the proportion of seedlings that survive to become plants from *t*0 to *t*1 in each subplot *j*. We calculated the sum of seedlings in spring and fall *t*0 (*R*sum_*j*,*t*0_) of a subplot and divided that number by the number of new individuals at *t*1 (*Ni*_*j*,*t*1_) in the same subplot.

(12) $$B_{j}={\mathrm{Ni}}_{j,t1}/{R\mathrm{sum}}_{j,t0}.$$

We calculated the log size distribution of new plants η which is defined as the normally distributed size of individuals that entered the continuous plant stage in year *t*1 (Equation 12). We used the mean ($\log_{e}\left({\hat{\eta }}_{t1}\right)$) and SD (σ_η_) of this size in our final parametrization of the IPM.

(13) $$\left(\eta_{i,t1}\right)\sim \text{Normal}\;\left(\log_{e}\left(\left({\hat{\eta }}_{t1}\right),\sigma_{\eta }\right)\right).$$

Our main analyses pool data across years for each species to have higher sample size (Table 2) and power to address our focal questions about climate and land management effects on demography. We also show results for separate IPMs for each yearly transition (2018–2019, 2019–2020, 2020–2021, 2021–2022) in Appendix S1: Figures S4–S6; these patterns were qualitatively similar to those using the pooled data approach.

**TABLE 2:** Shown is the pooled sample size of each species in each treatment combination.

Species	Ambient grazing	Ambient mowing	Future grazing	Future mowing
*Bromus erectus*	1141	1079	1075	1353
*Dianthus carthusianorum*	160	432	616	736
*Plantago lanceolata*	572	380	272	236
*Scabiosa ochroleuca*	424	400	288	428
*Tragopogon orientalis*	492	228	580	356

### Effects of treatments on vital rates

Separately for each species and continuous vital rate (survival, growth, reproduction probability, and number of seeds), we tested whether size, climate, management, and the according interactions had an influence on the vital rate using a maximum likelihood approach. We included a random term “plot” nested within climate to account for the nested design of the GCEF. Further, we calculated the mean and SD for each species and discrete vital rate (recruitment, establishment, and size distribution of new plants) for the effect of climate, management, and the interaction of climate and management.

To calculate the main effect of climate for each species and vital rate, we calculated the mean parameter for ambient and for future climate across the two land management types. For example, for the vital rate survival, we took the vital rate parameters extracted from the statistical model and calculated the mean across the respective land management treatment levels (e.g., survival parameter for ambient climate treatment is the mean of the parameters extracted for ambient grazing and ambient mowing). Likewise, to calculate the main effect of management for each species and vital rate, we calculated the mean for grazing and for mowing across the two climate treatments. Models for each vital rate were built only to provide the treatment‐specific parameters that are fed into the IPM machinery.

### Integral projection model

For each species, we created an IPM based on the vital rate models described before to calculate the combined and separate effect of climate and management on the long‐term population growth (λ). In the IPM, we calculate all possible transitions from *z* to *z*′. The change in number of plants from one year to the other is described by:

(14) $$n\left(z^{\prime },t1\right)=M\left(t0\right)B\eta \left(z^{\prime }\right)+\int_{L}^{U}S\left(z\right)G\left(z^{\prime },z\right)+P\left(z\right)F\left(z\right)\theta_{f}B\eta \left(z^{\prime }\right)n\left(z,t0\right)\mathrm{dz},$$

where the vector *n*(*z*′, *t*1) is the number of plants at size *z*′ at time *t*1. In this function, the first term represents the recruitment of spring seedlings entering the adult plant class. This is based on the number of spring seedlings at *t*0, *M*(*t*0), the seedling establishment, *B*, and the size distribution of new plants η(*z*′). The second kernel (or surface) describes the growth and survival of plants from *t*0, *n*(*z*, *t*0) to *t*1, *n*(*z*′, *t*1). It is defined as an integral with the upper limit *U*, which is the biggest size observed and the lowest size *L*, observed for the species in our study. The integrals were evaluated across 200 equally spaced size bins using the midpoint rules as a 200 × 200 matrix.

The recruitment of spring seedlings from one year to the next is described by:

(15) $$M\left(t+1\right)=\int_{L}^{U}P\left(z\right)F\left(z\right)\theta_{s}\mathrm{dz}.$$

As we could not distinguish if a new plant in April originated from seedlings found in fall or spring, we use the same establishment estimate for seedlings (*B*) twice in the model.

### Effects of treatments on population growth rate

To quantify uncertainty in estimates of λ for each species, we bootstrapped our data a thousand times and calculated the λ each time. To test for significant differences in λ between treatments and treatment combinations for each species, we used permutation (randomization) tests (*N* = 1000 permutations).

### Effects of flowering phenology and species indicator values on effect size of management and climate treatments on λ

To quantify the preference of a species for a specific management or climate treatment, we calculated an effect size of the grazing treatment on λ by subtracting λ in the mowing from λ in the grazing and respectively for the climate treatment (ambient λ − future λ). Therefore, positive effect sizes show a species preference for the grazing or ambient climate treatment, whereas, negative effect sizes show a species preference for the mowing or future climate treatment.

To test whether flowering phenology (i.e., flowering start month, duration of flowering) influenced the effect size of land management and climate on λ, we ran simple linear models using the effect sizes as response variables and the values of flowering duration and mean flowering start month as explanatory variables. Because flowering start and end month differed slightly between treatments for some species, we calculated the mean starting month of flowering and the mean end month of flowering for each species in each climate and management treatment and used these values to calculate flowering duration.

### Sensitivity of λ to each parameter

We calculated sensitivities of λ to each parameter in the IPM for each species and treatment. We extracted parameters for each species and treatment main effect and combination from the models described in the sections before. We then permuted each parameter individually by 0.001 and measured the change in λ.

### Life table response experiment

In order to decompose the influences of vital rates on the difference in λ, across treatment combinations, we conducted LTREs for each pairwise treatment combination. The difference in λ, Δλ, is:

(16) $$\Delta \lambda =\lambda^{\text{treatment combination}\;1}-\lambda^{\text{treatment combination}\;2}.$$

The contribution of each vital rate to Δλ, ${\tilde{\delta \;}}^{\alpha i}$, is:

(17) $${\tilde{\delta \;}}^{\alpha i}=\sum_{i}^{14}\left(\alpha_{i}^{\text{treatment combination}\;1}-\alpha_{i}^{\text{treatment combination}\;2}\right)\;\frac{\partial \lambda }{{\partial \alpha }_{i}}.$$

Here, α_*i*_ is one of the 14 parameters that are included in the IPMs, while the sensitivity of λ to each vital rate is defined as $\frac{\partial \lambda }{{\partial \alpha }_{i}}$. Vital rates will have a strong contribution to the observed difference in λ between treatment combinations if the vital rates change strongly in magnitude between the two treatment combinations and/or if λ is sensitive to changes in that vital rate. To facilitate the understandability, we combined LTRE results with respect to five demographic processes (survival, growth, reproduction, recruitment, and establishment; Table 3) and we scaled ${\tilde{\delta \;}}^{\alpha_{i}}$ to 1 (to display proportional influence).

**TABLE 3:** All vital rates and parameters that were used and extracted to calculate the integral projection model.

Vital rates	Abbreviation	Life‐cycle stage	Parameters	Distribution	LTRE stage
Plant survival	*S*	Survival	Intercept (int), slope	Bernoulli	Survival
Plant growth	*G*	Growth	Intercept (int), slope, SD	Normal	Growth
Reproduction probability	*P*	Reproduction	Intercept (int), slope	Bernoulli	Reproduction
No. seeds per reproductive plant	*F*	Reproduction	Intercept (int), slope	Poisson	Reproduction
Fall recruitment	θ_*f*_	Recruitment	Mean		Recruitment
Spring recruitment	θ_*s*_	Recruitment	Mean		Recruitment
Seedling establishment	*B*	Establishment	Mean		Establishment
Size distribution of new plants	η	Establishment	Mean, SD		Establishment

Abbreviation: LTRE, life table response experiment.

### Analysis

All analysis were carried out in R (R Core Team, 2022). Figures were made using the ggplot2 package (Wickham, 2016). For the mixed‐effect models, we used the package lme4 (Bates et al., 2015). The IPMs were run and calculated using the ipmr package (Levin et al., 2021). Other packages used were: openxlsx (Schauberger & Walker, 2021) to load the xlsx files into R, dplyr (Wickham et al., 2022) for structuring data and piping, and the package parallel (R Core Team, 2022) to decrease the running time of R scripts.

## RESULTS

### Quasi extinction

The following six species went quasi extinct over the course of the experiment: *Anthoxanthum odoratum*, *Crepis biennis*, *Lotus corniculatus*, *Lychnis flos‐cuculi*, and *Trifolium pratense*. While there is too small of a sample size (*N* = 11 species) for statistical analysis, it is notable that the species that went quasi extinct were also the ones that were less drought tolerant based on their Ellenberg values (Table 1) and that the first few years of our study were extraordinarily dry for our region (Boergens et al., 2020).

### Management effects on population growth rate

**FIGURE 2:**
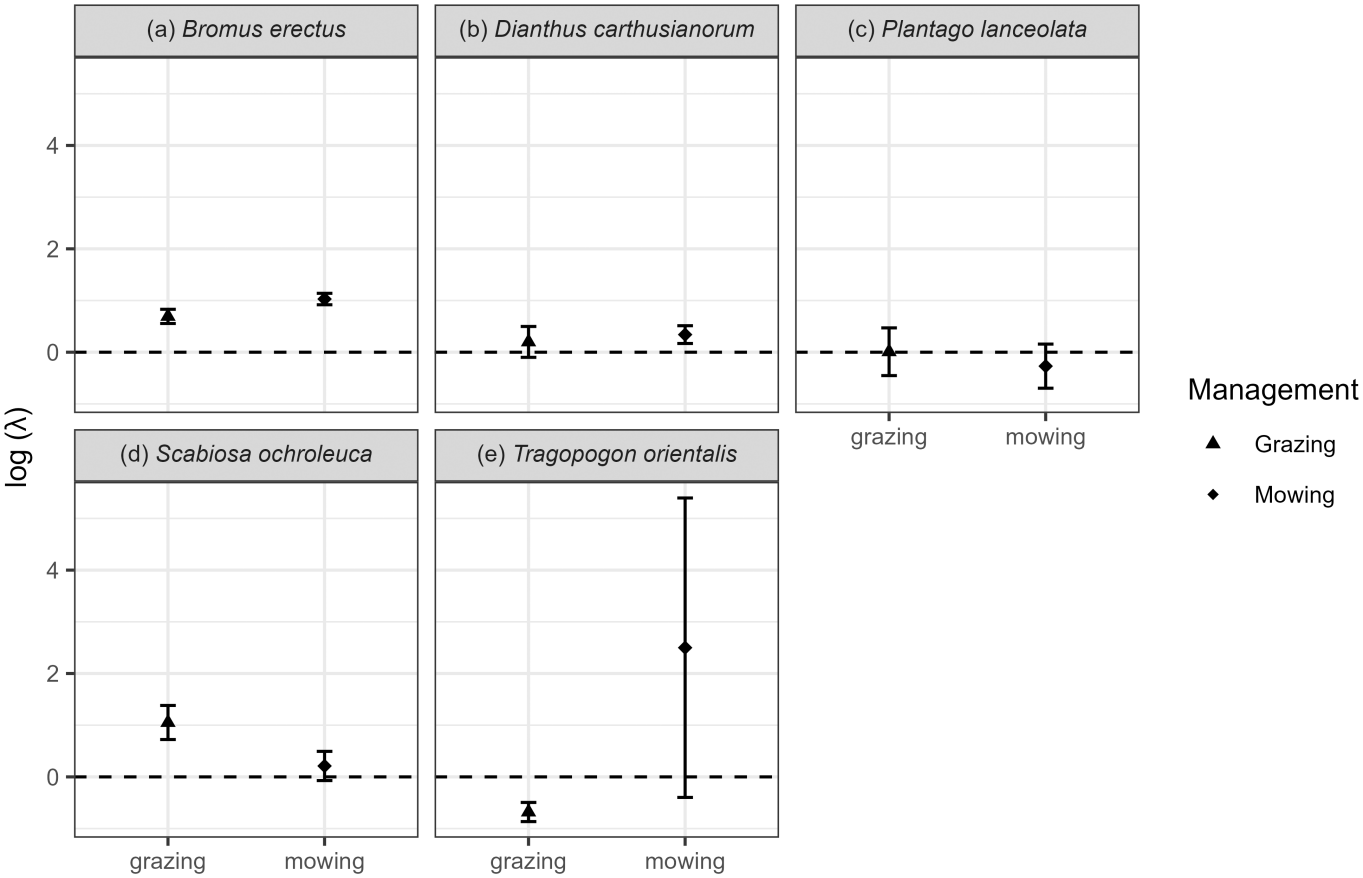
Effect of land management type (grazing and mowing) on log population growth rate (λ) of the five study species (a–e). Displayed is the mean logged λ of 1000 bootstraps and the SD.

For all species, except *T. orientalis* and *P. lanceolata*, the population growth rates (log λ) were projected to grow (log λ > 0) in pastures and meadows. *T. orientalis* was projected to decline in log λ in the grazing treatment and *P. lanceolata* had log λ values close to zero (stable populations) in both management types. For three species, *T. orientalis*, *B. erectus*, and *Scabiosa ochroleuca*, there was a significant main effect of management type; two species had higher λ in mowing compared with grazing management (permutation tests *B. erectus p* = 0.014, *T. orientalis p* = 0.02, Figure 2), and one species had higher λ in the grazing treatment (*S. ochroleuca p* = 0.048, Figure 2). No other species showed any significant main effect of management on λ (Figure 2).

Unfortunately, we could not test whether grazing resistance or mowing resistance indicator values correlated with the effect size of log λ due to the quasi extinction of six species, along with missing indicator values for two of the five remaining species. Flowering phenology (flowering duration and flowering start) did not explain the response of plant species to the land management types (Appendix S1: Figure S2).

### Climate effects on population growth rate

**FIGURE 3:**
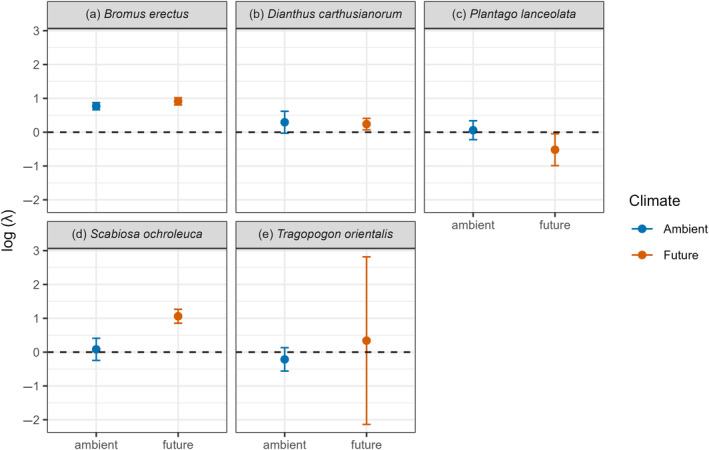
Effect of climate (ambient and future) on log population growth rate (λ) of the five study species (a–e). Displayed is the mean logged λ of 1000 bootstraps and the SD.

Three species (*B. erectus*, *Dianthus carthusianorum*, and *S. ochroleuca*) were projected to have an increasing population growth (log λ > 0) under both climate treatments. The population growth rate of *T. orientalis* was projected to increase under future climate and to decline under ambient climate. The population growth rate of *P. lanceolata* was projected to decline under future climate conditions (log λ < 0) but had a log λ close to zero (stable population) in the ambient climate. *S. ochroleuca* had higher log λ under future compared with ambient conditions (permutation test, *p* < 0.01) and all other species showed no significant differences in log λ between the climate treatments despite some shifts in the direction of log λ (Figure 3a–e). Flowering phenology did not explain the responses of species to the climate treatments (Appendix S1: Figure S3).

### Interactive effect of climate and land management type on population growth rate

**FIGURE 4:**
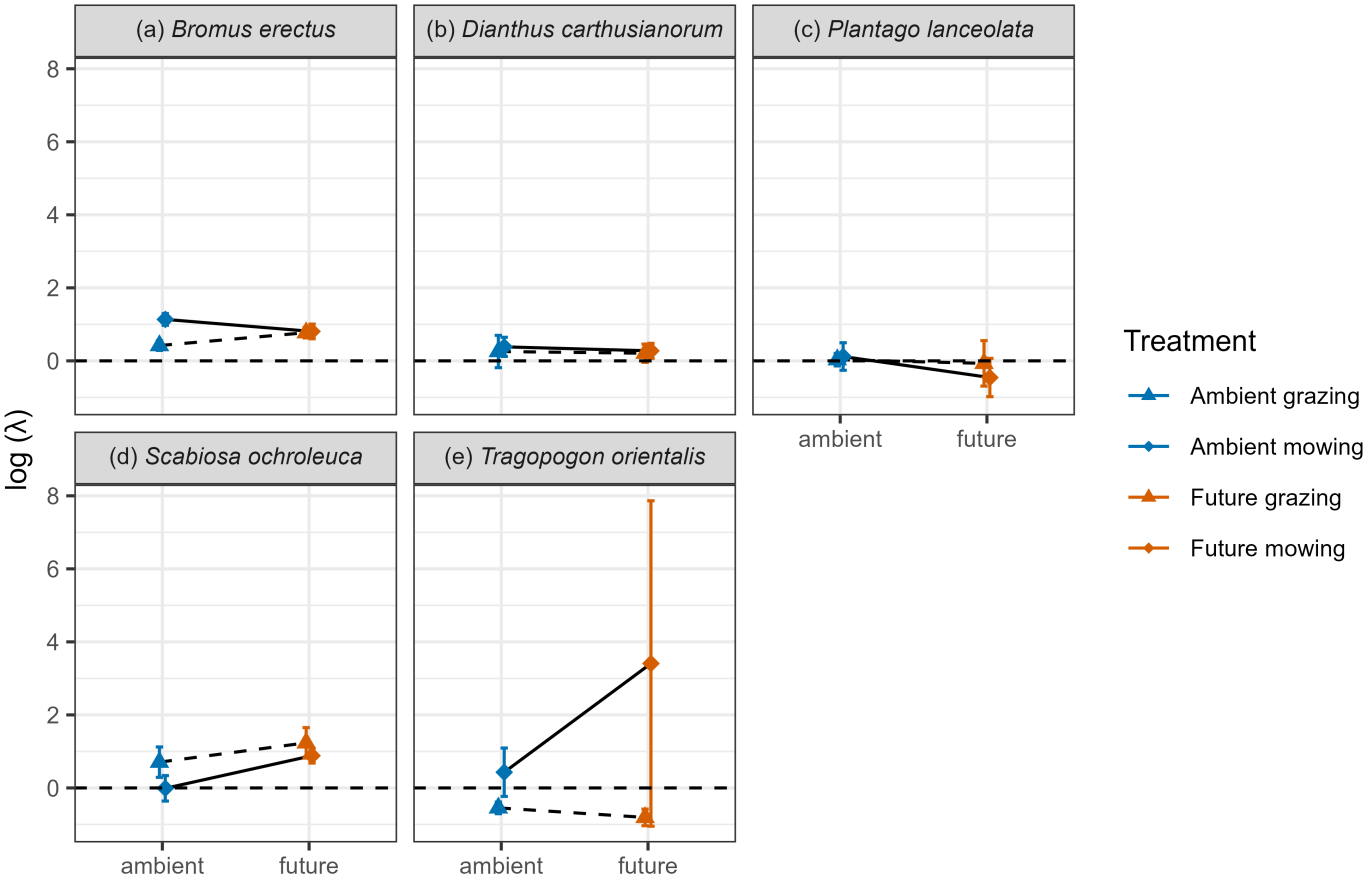
Effect of climate (ambient and future) and land management type (grazing and mowing) on log population growth rate (λ) of the five study species (a–e). Displayed is the mean logged λ of 1000 bootstraps and the SD.

**FIGURE 5:**
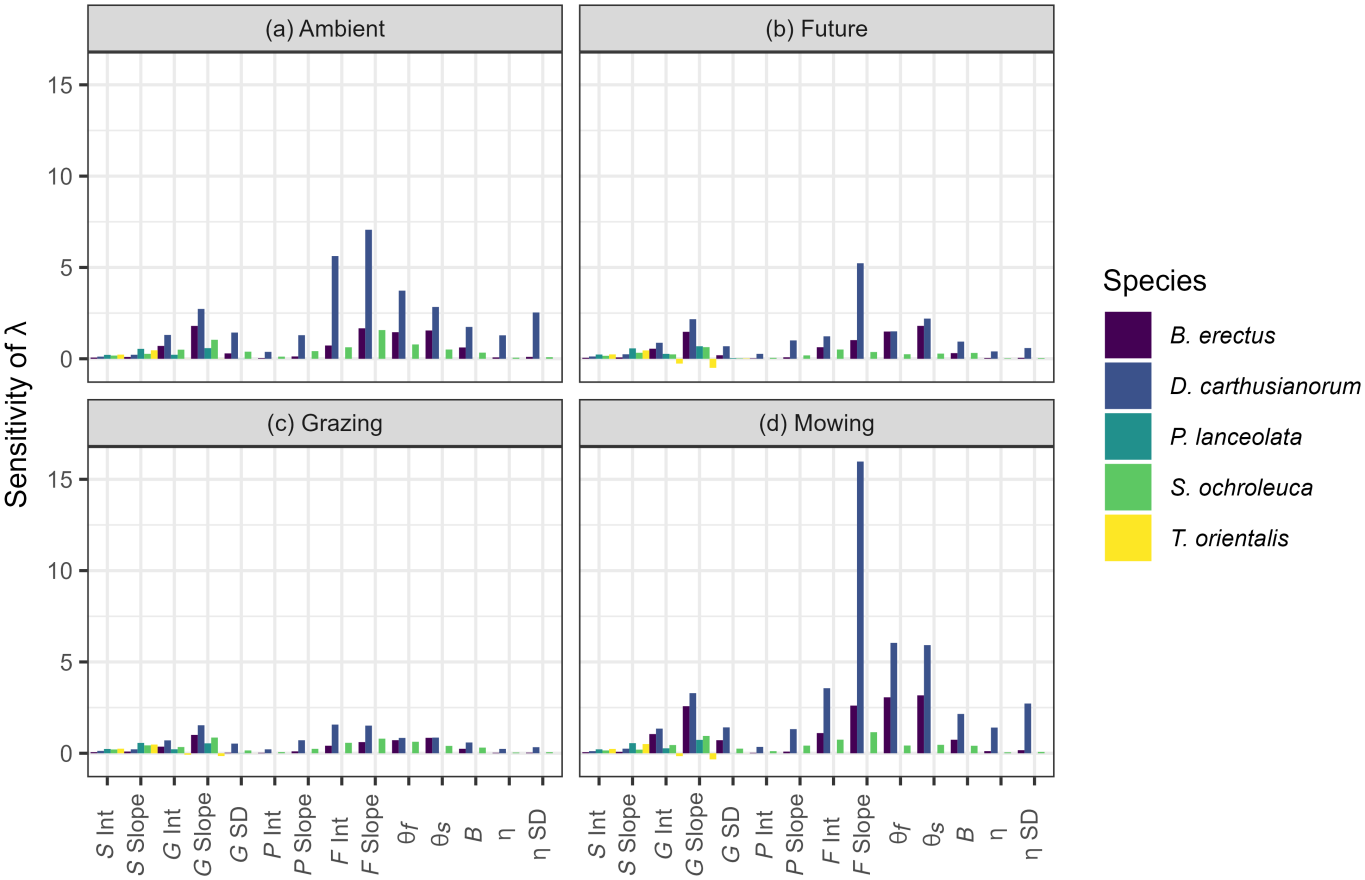
Sensitivity of pairwise treatment combinations for every vital rate included in the integral projection model. Each species is displayed in a different color. The *x*‐axis labels represent each parameter extracted from the vital rates: *S* Int = intercept of the survival model, *S* Slope = slope of the survival model, *G* Int = intercept of the growth model, *G* Slope = slope of the growth model, *G* SD = SD from the growth model, *P* Int = intercept from the reproduction probability model, *P* Slope = slope from the reproduction probability model, *F* int. = intercept of the number of seed model, *F* Slope = slope of the number of seeds model, θ_*f*_ = mean recruitment fall, θ_*s*_ = mean recruitment spring, *B* = seedling establishment, η = size distribution of new individuals, η SD = SD of size distribution of new individuals.

Several species showed evidence that climate and management type interactively influenced population growth rate log λ. *S. ochroleuca* had a log λ close to zero in the ambient climate and shifted to a clearly positive log λ under future climate conditions in the mowing treatment only (future mowing > ambient mowing; permutation test, *p* = 0.029), meaning that this species population size was projected to increase in meadows under future climate conditions. *B. erectus* populations were growing under all conditions but had significantly higher log λ values in the mowing than in grazing treatment but only under ambient climate conditions (permutation test, *p* < 0.001) (Figure 4a). The log λ of *T. orientalis* was significantly higher in mowing compared with grazing management only in the ambient climate treatment (permutation test, ambient grazing < ambient mowing, *p* = 0.036 Figure 4e). For *P. lanceolata*, the lines in the interaction plots crossed but treatment combinations were not significantly different from each other (Figure 4c). *P. lanceolata* showed stable populations in all treatment combinations (log λ close to 0), except in future mowing for which the population was projected to decline (log λ = −0.46). Treatment combinations were not significantly different from each other for *D. carthusianorum* (Figure 5b), and positive population growth was projected for all treatment combinations (log λ > 0).

### Sensitivity

Consistently across all species and treatments, log λ was most sensitive to changes in vital rates related to reproduction (number of seeds and recruitment) and growth parameters (Figure 5). However, the magnitude of sensitivity of log λ to specific vital rates differed between treatments and species. For example, log λ of *D. carthusianorum* was highly sensitive to changes in the vital rate number of seeds in the mowing treatment, indicating that changes in the seed output of this species would result in the largest changes to population growth.

### Life table response experiment

**FIGURE 6:**
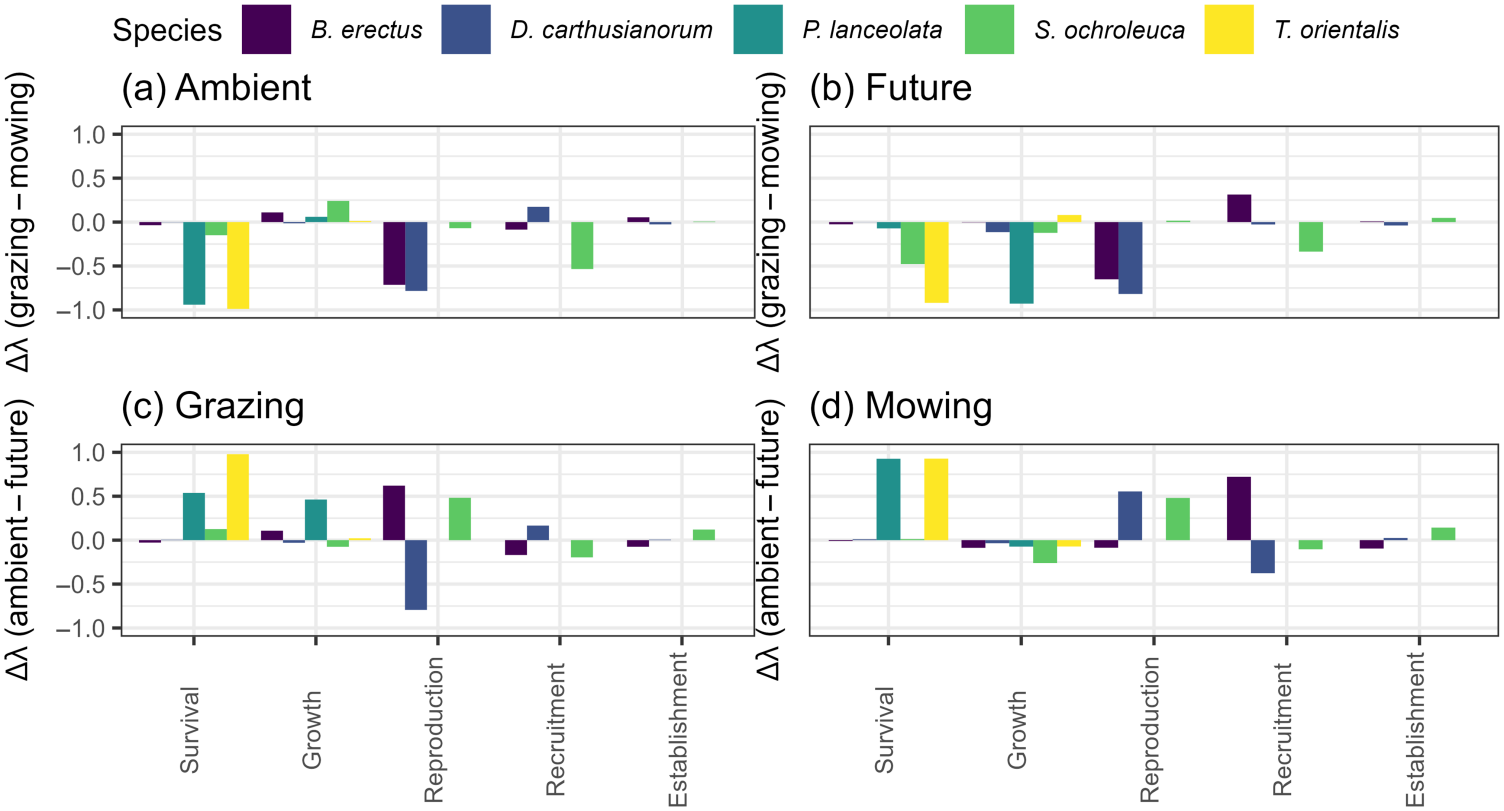
Results for the Life Table Response Experiment. Each species is displayed in a different color. (a) Ambient climate treatment and the effect of the two land management types on the parameters. (b) Future climate treatment and the effect of the two land management types on the parameters. (c) Grazing land management type and the effect of climate on the parameters. (d) Mowing land management type and the effect of climate on the life stages. For a better overview, we summed the parameters according to their corresponding live stage (survival, growth, reproduction, recruitment, establishment).

The higher log λ of *B. erectus* in ambient mowing compared with the ambient grazing treatment combination was primarily due to increases in reproduction (Figure 6a). Similarly, the higher log λ of *S. ochroleuca* in future mowing compared with ambient mowing was primarily due to increases in the reproduction of this species (Figure 6d). The higher log λ of *T. orientalis* in ambient mowing compared with the future mowing treatment was primarily due to increases in plant survivorship (Figure 6a). *D. carthusianorum* and *P. lanceolata* did not show any differences in their log λ across treatments.

## DISCUSSION

Our study aimed to examine whether plant species interactively respond to climate and land management types in their demography and population dynamics. We studied this question across many plant species that are common in European temperate grasslands. However, in the early years of our study, six of our 11 focal species went quasi extinct from the experiment, likely due to the extreme drought conditions that occurred in our region in 2018 and 2019. This is becoming an increasing phenomenon in experimental global change research—even the control treatments experience extreme climate events because these events are now more common in our rapidly changing world (IPCC, 2014; Jentsch et al., 2007; Korell et al., 2024; Orlowsky & Seneviratne, 2012; Tebaldi et al., 2006). We found no consistent support for our expectation that plants would perform better in ambient compared with future climate conditions, likely because most species that persisted in the experiment were the more drought tolerant ones. We were left with limited power to test for the role of flowering phenology in predicting the responses of the species population growth rate (λ) to the treatments, but still found no support for the idea that plants with early flowering start dates and/or longer flowering durations perform better in the mowing management or future climate treatments. In accordance with our expectations, we found evidence of interactive effects for multiple species, suggesting that the optimal land management will change in the future with climate change.

The first year of our demographic study, 2018, coincided with the driest summer in Germany since the start of recording (Schuldt et al., 2020). This drought had dramatic and long‐lasting effects on plant communities in Germany and on the plant communities in our GCEF experiment (Korell et al., 2024). Many of the species that went quasi extinct were those that are indicative of moist soil conditions. Three of the five species that remained are indicative of drier habitat conditions and commonly occur in semidry or dry meadows (*B. erectus*, *D. carthusianorum*, and *S. ochroleuca*), or are known to be highly generalized with regard to the moisture of the habitat (*P. lanceolata*). This shows that in future it will be difficult for plants adapted to wetter conditions to persist in their current habitats.

Only one of our remaining species, *T. orientalis*, is indicative of moist to moderately dry soil conditions (Bomble, 2013; Ellenberg et al., 1991; Sebald et al., 1990) and managed to persist in our study. However, this species performed overall poorly, and our models did not project this population to grow in any treatment combination. If we had been able to study the demography of more typical species from moister grassland communities as we initially planned, we may have supported our hypothesis that future climate would harm plant populations. Our study highlights the importance of historical legacies in shaping experimental results and support projections that communities may shift toward more drought‐tolerant species under future climate conditions (Belovsky & Slade, 2021; Craine et al., 2013).

We found evidence for our hypothesis that interactive effects of treatments on λ would be common in our study and that these patterns would be species‐specific. For *B. erectus*, we found that mowing management is better than grazing in ambient climate, but that there was no effect of management type on λ in future climate (see also Lemmer et al., 2021). This is because higher rates of reproduction in the mown treatment were counteracted by lower rates of recruitment under future climate. It is shown that *Bromus* seeds are highly sensitive to drying out (Bertiller et al., 1996; Soriano & Sala, 1986), and this might explain why future climate conditions limit recruitment and thus also the beneficial effect of the mowing management. Grazing happens before mowing in our system, and sheep eat the flowers of *B. erectus* before they can produce ripe seeds, whereas, most reproductive individuals have fertile seeds before the mowing occurs.

*T. orientalis* had a significant higher λ in mowing compared with grazing management in the ambient climate, but this effect is not significant under future climate conditions. However, this species had extremely high variation in λ in the future mowing treatment due to very high seed production of a few plants in this treatment combination. Our results showing a preference for mowing over grazing management are in line with several other studies showing negative effects of grazing on plant population dynamics (Hansen & Wilson, 2006; Jacquemyn et al., 2012; van der Meer et al., 2014). For example, in *Primula veris*, grazing was found to decrease λ, primarily through its negative effect on reproduction (Jacquemyn et al., 2012). It would be ideal if plant functional traits could predict species preferences for mowing and grazing treatments, given the difficulty of collecting performance data in field studies for single species. However, to date, this synthesis has been a difficult endeavor because no one trait correlates well to grazing versus mowing preference, and many traits and trade‐offs are involved (Cingolani et al., 2005; Klimešová et al., 2008).

*S. ochroleuca* performed worst (but still has a stable population) in the ambient mowing treatment combination due to lower reproduction. The population was projected to grow in the same mowing management treatment under future climate. One possible mechanism that could explain why the future climate treatment amplifies the positive effect of mowing on λ of *S. ochroleuca* is that this drought‐tolerant species has an advantage over its competitors in future climate. The first mowing event occurs early in the summer, just before the summer drought in the future climate treatment. *S. ochroleuca* may be better able to regrow and reproduce in these dry conditions than its competitors.

The population growth rate of the remaining two species (*D. carthusianorum* and *P. lanceolata*) did not show any significant response to climate change and management nor indication for interactive effects of the two treatments. *P. lanceolata* is a rosette plant close to the ground, and therefore experiences limited direct harm from both management types, and many benefits from having competitor plants removed. *D. carthusianorum* is similarly known to benefit from both types of extensive land management (Poschold et al., 1996). Extensive grassland management through grazing and/or mowing are practices that provide economic value as well as value for preserving biodiversity (Tälle et al., 2016). Across all of our study species, we can conclude that future climate might cause the local extinction of species that are currently common in these grassland communities. Further, in ambient climate, species often show distinct preferences for different management types (grazing vs. mowing). We observed that these preferences become less clear under future climate conditions.

In demographic studies of both plants and animals, reproductive output is known to be skewed across individuals, with only a few individuals producing many offspring (van Daalen & Caswell, 2020). The ability to distinguish deterministic treatments effects from stochasticity among individuals would require much higher sample sizes than is available in our GCEF experiment. While there are many advantages of conducting multispecies demographic studies like this one and of conducting demographic research in a controlled experimental setting, one distinct disadvantage is the inability to have the sample size required to adequately detect small changes in sensitive vital rates (McMahon et al., 2019).

It was surprising to find in our LTRE that changes in reproduction and recruitment often contributed most to the observed responses of λ to management and climate. Usually in longer‐lived species, λ is highly sensitive to changes in survival (Kuss et al., 2008; Weppler et al., 2006) and, thus, even small changes in survival across treatments should contribute significantly to changes in λ. In our case, we found that λ is highly sensitive to reproduction. This might be due to the relatively high λ of our perennial species; species with high λ are often more sensitive to reproduction and recruitment than to survivorship (Franco & Silvertown, 2004; Ramula et al., 2008). Further, many of our species were able to maintain their survival across treatments but had dramatic changes in their reproduction.

## CONCLUSIONS

Our study is a first step toward answering important, yet unanswered, questions on how climate change affects population dynamics of grassland species, and how this effect is modified by different land management types. On the pessimistic side, more than half of our common grassland species went quasi extinct during the course of our study and have still not returned to our system, suggesting that it will be more difficult to create and maintain mesophilic grasslands composed of species with preference for moister soil conditions in the future. Our results are optimistic for the species that remained that were mostly drought‐tolerant species that are characteristic for dry or semidry grasslands. These species showed mostly persistent or growing populations in all of our treatment combinations. Interactive effects of land management and climate on plant population dynamics were common in our study, suggesting that land managers might have to alter their practices in response to our changing climate. Furthermore, based on our finding of high sensitivity of populations to changing reproduction, we suggest that management should carefully be timed outside of peak flowering and fruiting, so that as many species as possible can reproduce and persist in managed grasslands.

## AUTHOR CONTRIBUTIONS

The study was designed by Lotte Korell (leading) and Martin Andrzejak and Tiffany M. Knight (supporting). Fieldwork was designed and carried out by Lotte Korell and Martin Andrzejak (both leading). The data for the phenology of plants were collected by Carolin Plos (leading). Analysis was carried out by Martin Andrzejak (leading) with support of the other coauthors. The manuscript was written by Martin Andrzejak (leading) and Tiffany M. Knight and Lotte Korell (supporting). All authors commented and improved the manuscript.

## CONFLICT OF INTEREST STATEMENT

The authors declare no conflicts of interest.

## Supporting information


Appendix S1:

## Data Availability

Data are available in Figshare as follows: phenology data (Andrzejak, 2023), https://doi.org/10.6084/m9.figshare.22730708.v1; demography data (Andrzejak et al., 2023a), https://doi.org/10.6084/m9.figshare.21941603.v1; seed data (Andrzejak et al., 2023b), https://doi.org/10.6084/m9.figshare.21941618.v1. Code (Martin, 2024) is available on Zenodo at https://doi.org/10.5281/zenodo.14030219.
