# Supplementary material for: Changes in reproduction mediate the effects of climate change and grassland management on plant population dynamics
Source: Ecol Appl. 2024 Dec 8;35(1):e3063. doi: 10.1002/eap.3063 (PMC11737008; doi:10.1002/eap.3063)

Ecological Applications

Changes in reproduction mediate the effects of climate change and grassland management on plant population dynamics

Martin Andrzejak, Tiffany M. Knight, Carolin Plos, Lotte Korell

Figure S1 Layout of every GCEF subunit. The outer box is the subunit, the inner represents the area where experimental measurements are taken, and the green rectangle is the transect. The green bar indicates the location of the transect in which demographic data was collected for this study Subplots of the transect are not displayed as their position changes between transects.

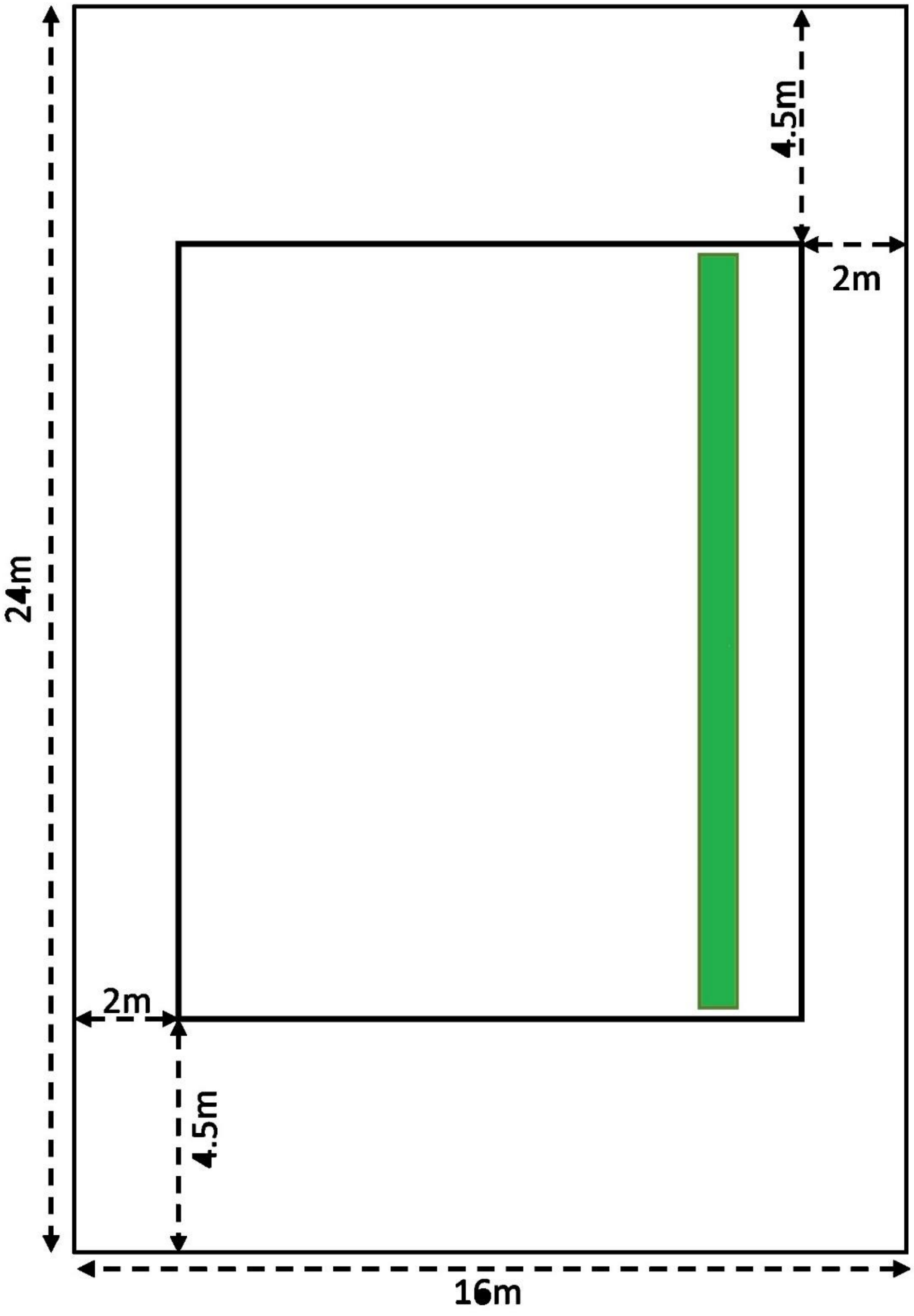

Table S1 Dates of the management events. The grazing event involved 20 sheep grazing one plot for 24 hours. The sheep were let on one plot in the morning of the day and the next day they moved to the next plot; grazing of all plots was completed during the range of dates in the table. The mowing event occurred on all plots in the mowing treatment in a single day.

Management event	Year	Date
Grazing	2018	30.04 - 11.05
Grazing	2018	11.06 - 22.06
Mowing	2018	11.06
Grazing	2019	6.05 - 17.05
Grazing	2019	10.06 - 21.06
Mowing	2019	11.06
Grazing	2020	11.05 - 22.5
Grazing	2020	22.06 - 03.07
Mowing	2020	08.06
Grazing	2021	10.05 - 21.05
Grazing	2021	14.06 - 25.06
Mowing	2021	14.06
Mowing	2021	06.09.2023
Grazing	2021	06.09 - 17.09

Table S2 The size information that was used to classify individuals as seedlings for each plant species in the study.

**Species**	**Maximum size as a seedling**
Anthoxanthum odoratum	< 4 leaves
Bromus erectus	1 ramet with maximum 3 leaves
Crepis bunnies	1 ramet with maximum 3 leaves
Dianthus carthusianorum	Size of rosette <0.25cm^2
Lotus corniculatus	Size of rosette <0.25cm^2
Lychnis flos-cuculi	No seedlings found
Medicago falcata	Size of rosette <0.25cm^2
Plantago lanceolata	1 ramet with maximum 3 leaves
Scabiosa ochroleuca	1 ramet with maximum 2 leaves
Tragopogon orientalis	Individual with maximum 3 leaves
Trifolium pratense	Individual with maximum 2 leaves

Figure S2 Relationship between the effect of management on λ (effect size λ grazing – λ mowing) plotted against a) the mean duration of flowering for each species and b) the mean start of flowering. Neither result was statistically significant (linear regression $P > 0.05$)

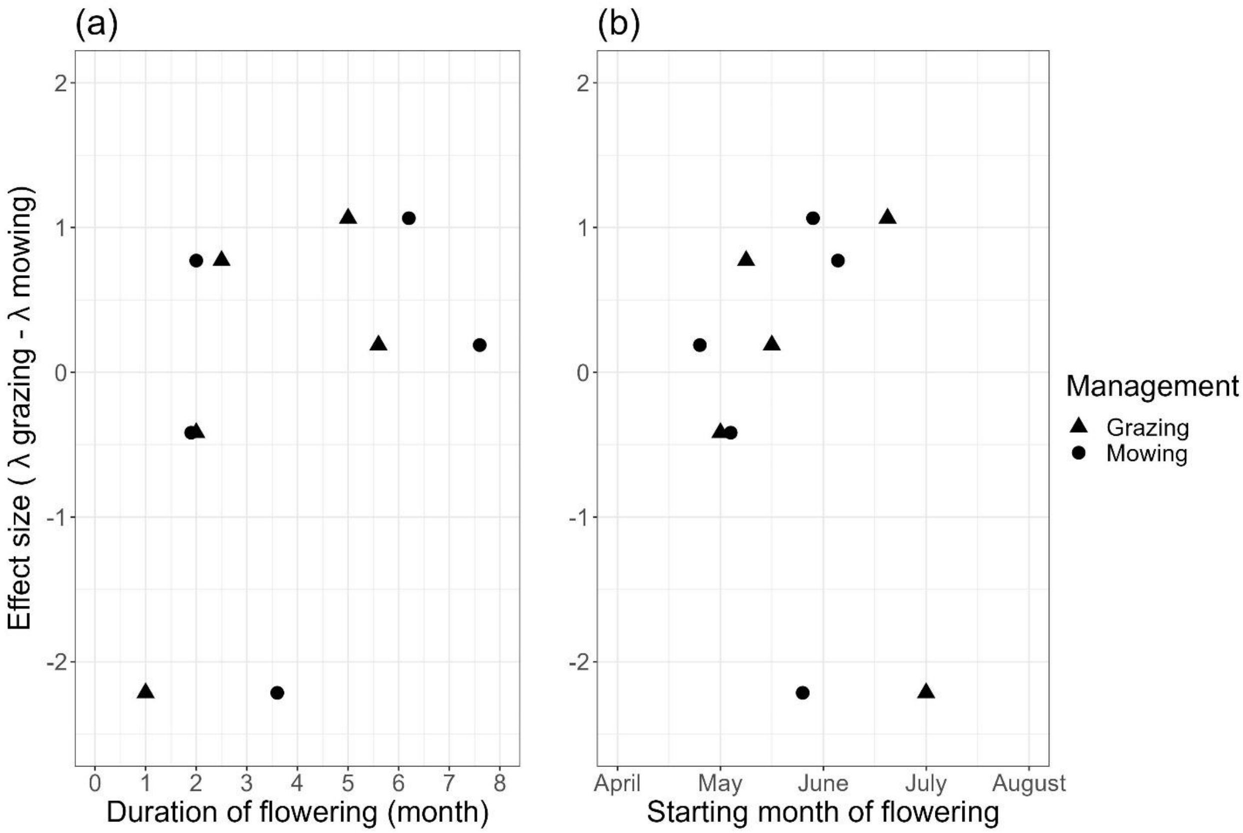

Figure S3 Relationship between the effect of climate on λ (effect size: λ ambient – λ future) plotted against a) the mean duration of flowering for each species and b) the mean start of flowering. Neither result was statistically significant (linear regression $p > 0.05$)

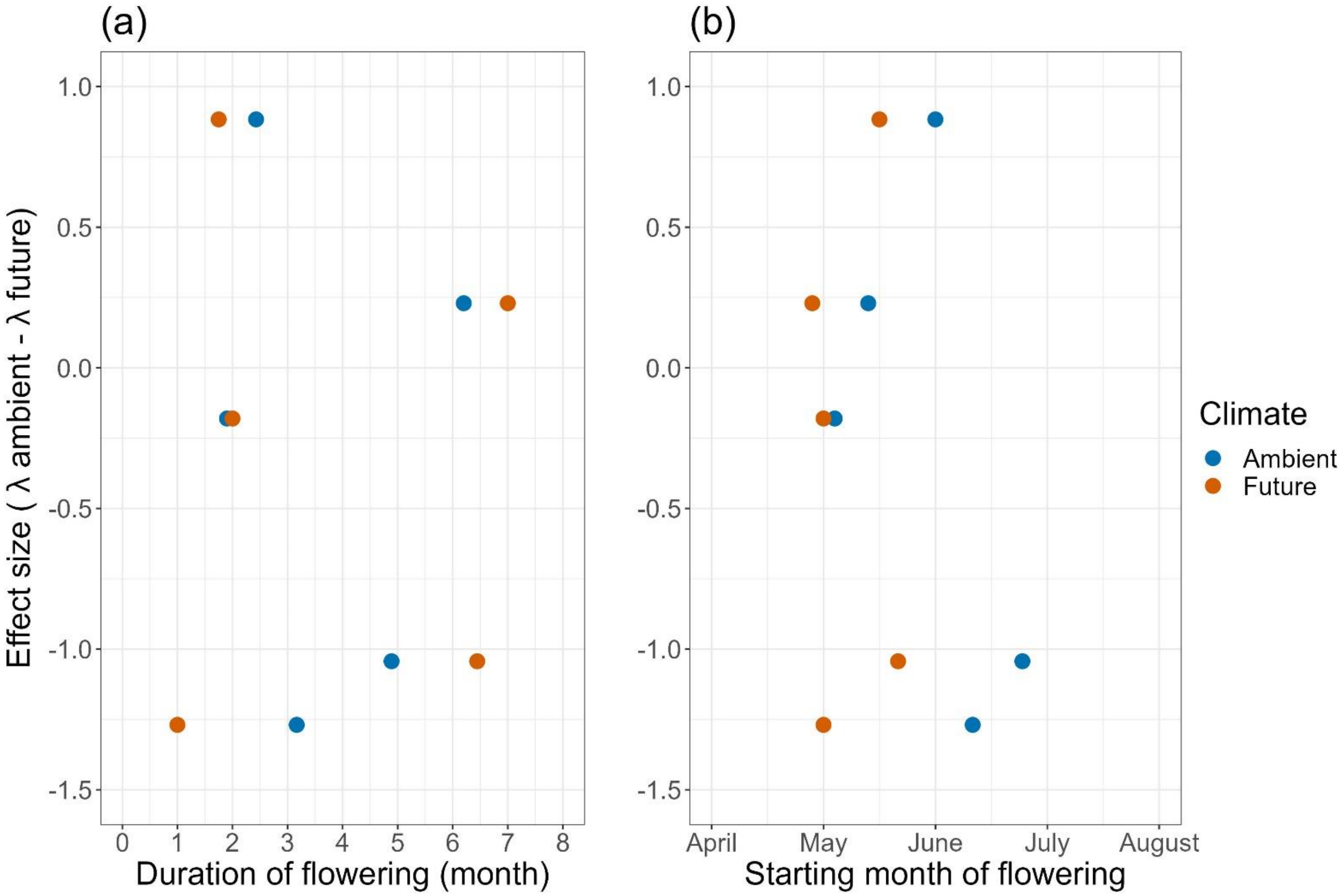

Figure S4 Log population growth rate (λ) for each species, year, and management treatment (mowing, grazing). Displayed is the mean λ and the standard deviation. Each year on the x axis stands for a transition: 2018 = 2018–2019, 2019 = 2019–2020, 2020 = 2020–2021, 2021 = 2021–2022. We were not able to calculate a λ for *T. orientalis* in some treatments and years due to low sample size.

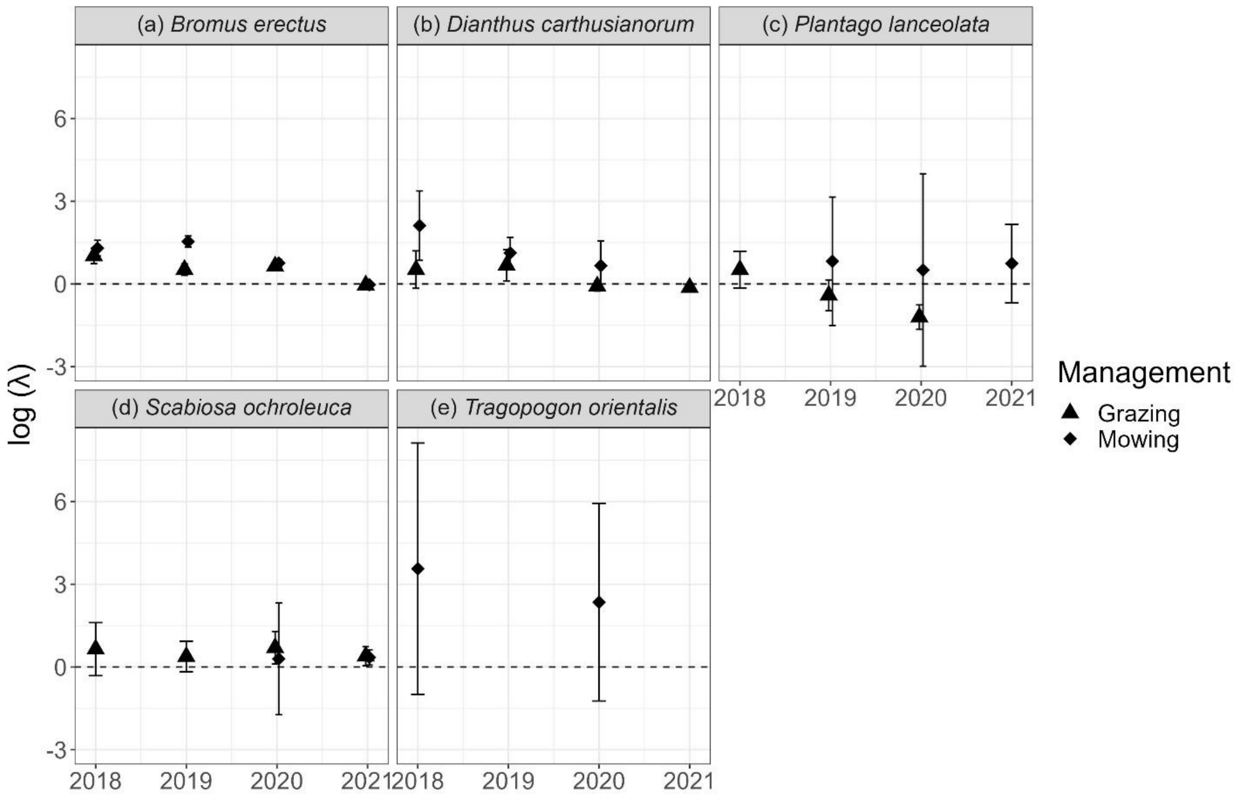

Figure S5 Log population growth rate (λ) for each species, year, and climate treatment (ambient, future). Displayed is the mean λ and the standard deviation. Each year on the x axis stands for a transition: 2018 = 2018 – 2019, 2019 = 2019 – 2020, 2020 = 2020 – 2021, 2021 = 2021 – 2022. We were not able to calculate a λ for *S. ochroleuca* and *T. orientalis* in some treatments and years due to low sample size.

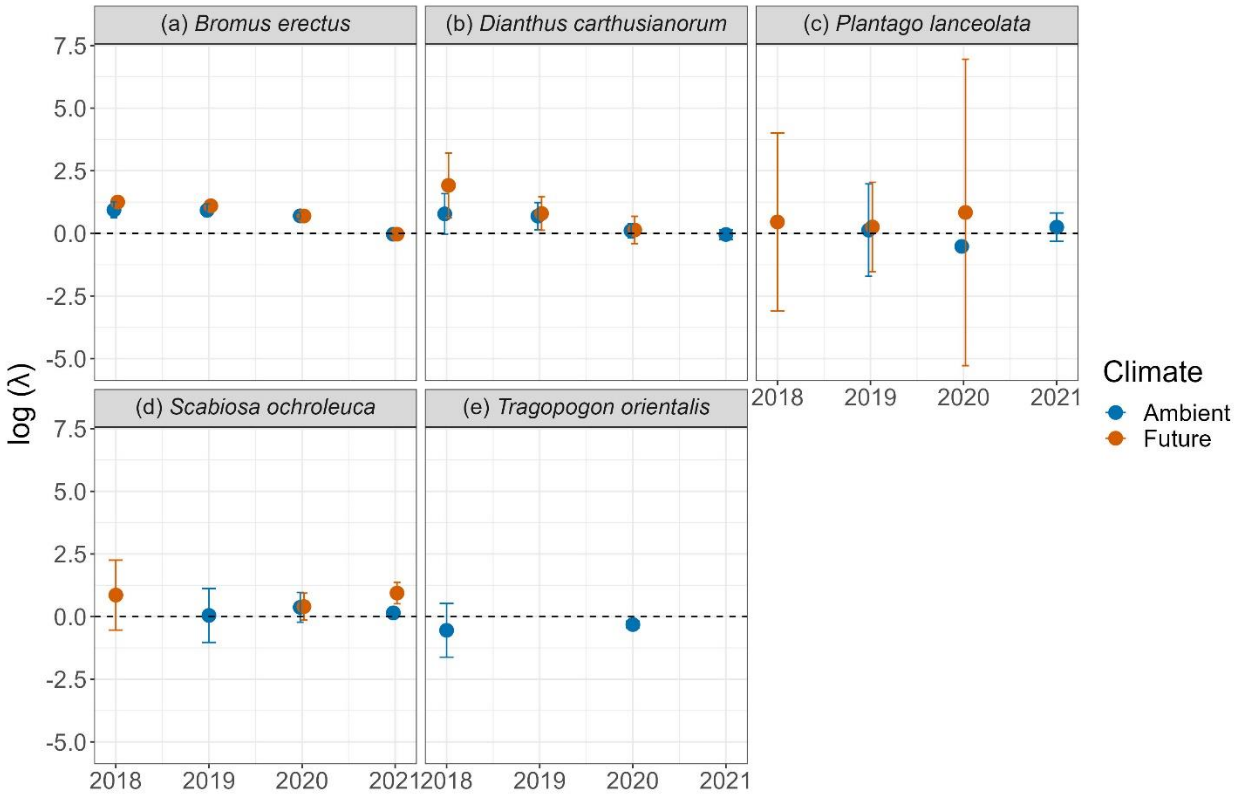

Figure S6 Log population growth rate (λ) for each species, year, and treatment combination (management and climate). Displayed is the mean λ and the standard deviation. Each year on the x axis stands for a transition: 2018 = 2018 – 2019, 2019 = 2019 – 2020, 2020 = 2020 – 2021, 2021 = 2021 – 2022. Missing points are due to low sample size and an inability to calculate λ.

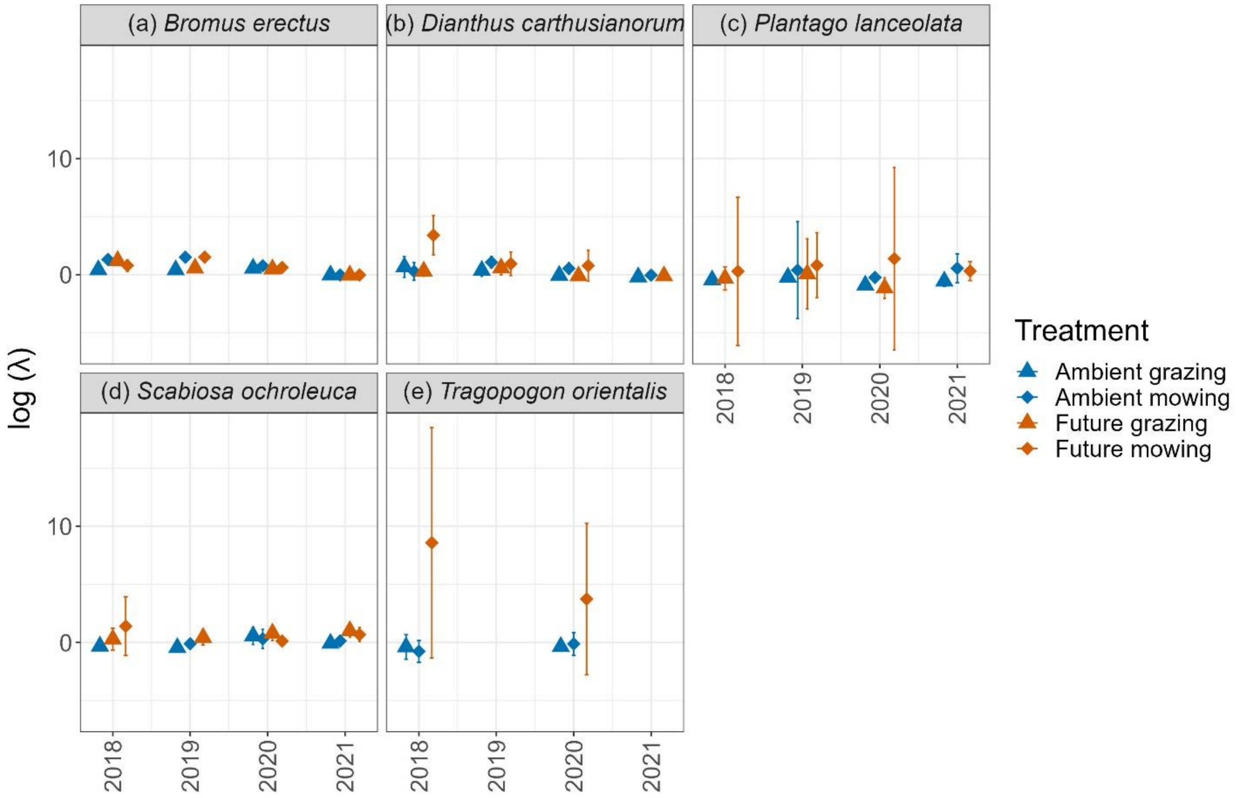

Figure S7 Number of seeds produced per individual plant plotted against the size of each reproductive individual for the species (a) *Bromus erectus*, (b) *Dianthus carthusianorum*, (c) *Plantago lanceolata*, (d) *Scabiosa ochroleuca* and (e) *Tragopogon orientalis*. Different treatment combinations are indicated with different shapes and colors.

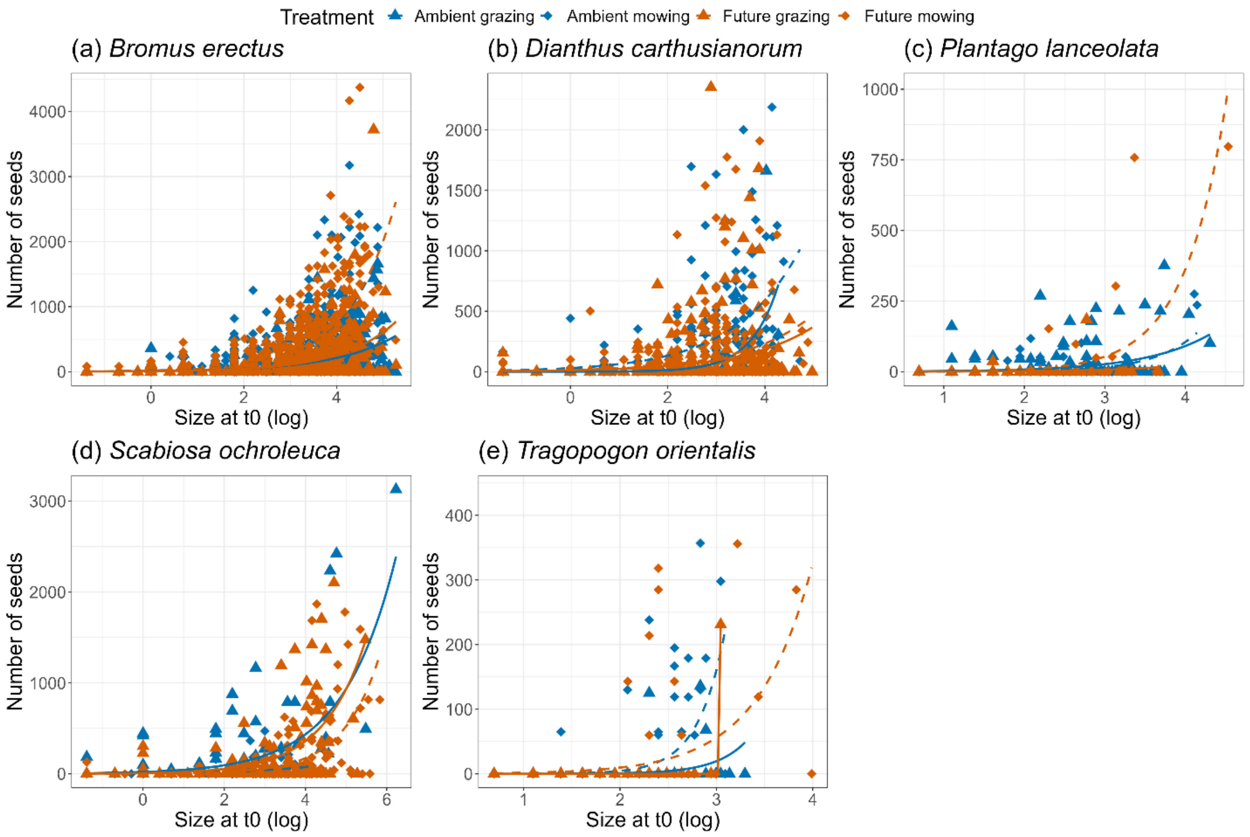

Figure S8 Proportion of seeds that become seedlings in the spring (i.e., spring recruitment in each treatment combination for (a) *Bromus erectus* (b) *Dianthus carthusianorum* (c) *Plantago lanceolata* (d) *Scabiosa ochroleuca* (e) *Tragopogon orientalis*. The error bars indicate the standard deviation.

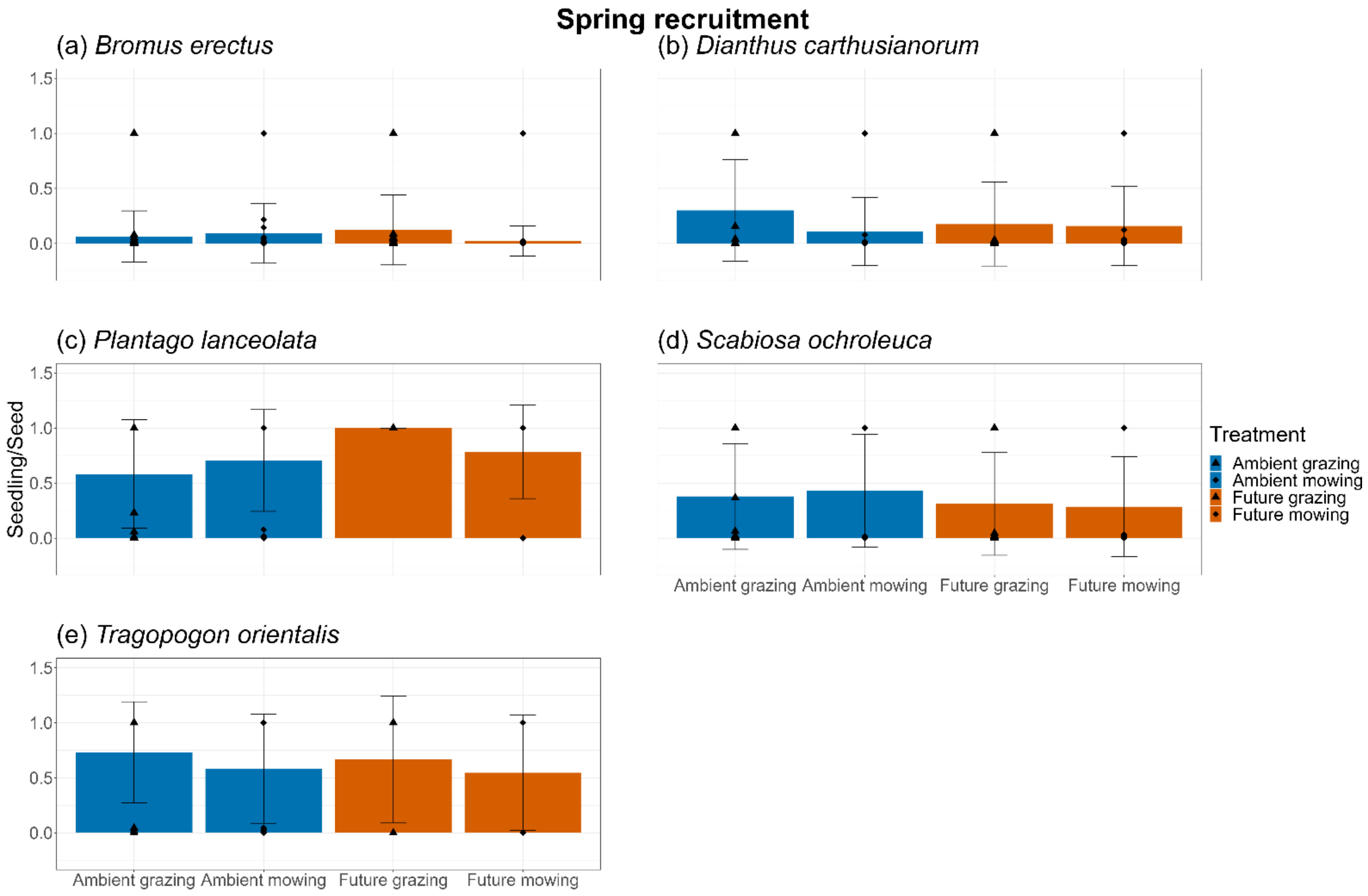

Figure S9 Proportion of seeds that become seedlings in the fall (i.e., fall recruitment) in each treatment combination for (a) *Bromus erectus* (b) *Dianthus carthusianorum* (c) *Plantago lanceolata* (d) *Scabiosa ochroleuca* (e) *Tragopogon orientalis*. The error bars show the standard deviation.

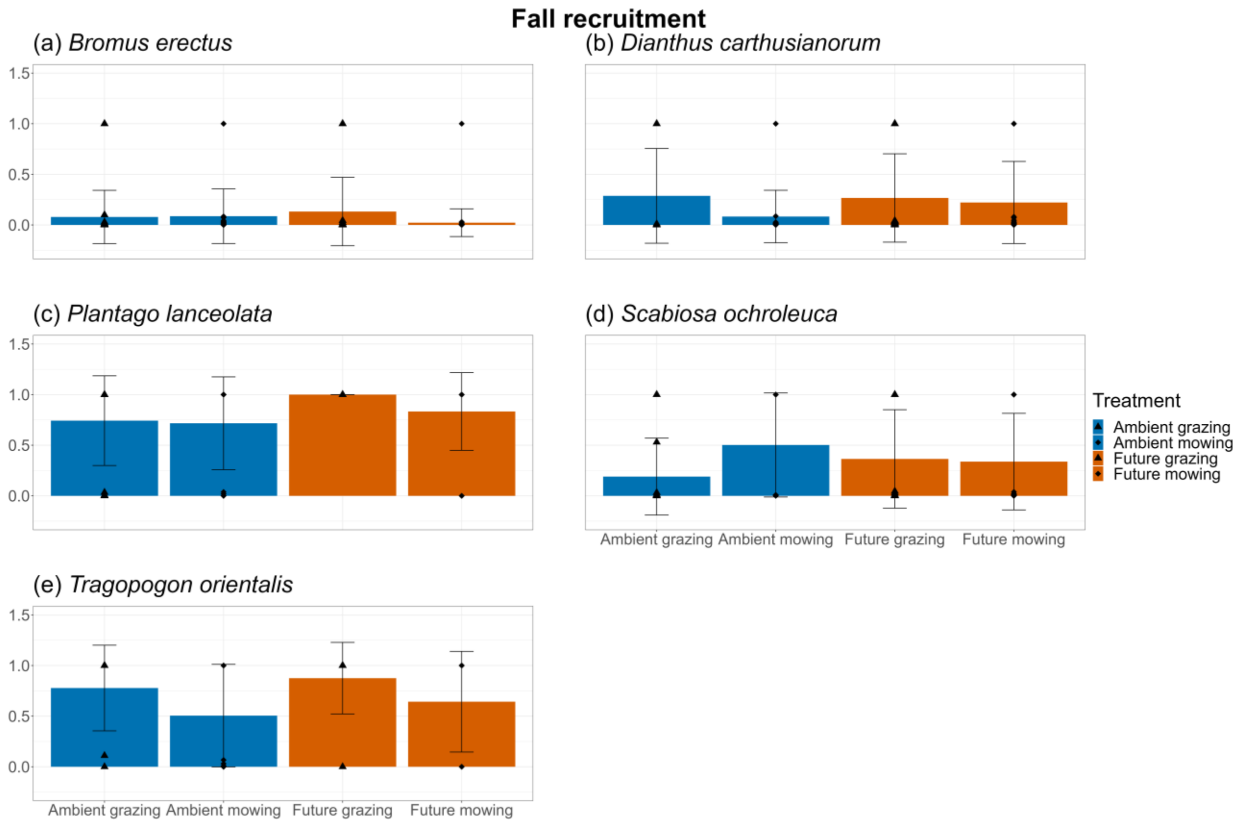

Table S3 Relationship between plant size and vital rates (survival, growth, flower probability, number of seeds). Presented in the table are the P-values and measure of fit for each vital rate model and species.

Species	Survival		Growth		Reproduction probability		Number of seeds	
	p	deviance	P	r2	P	Deviance	P	Deviance
B. erectus	<0.05	1092.37	<0.05	0.75	<0.05	2084.43	<0.05	212652.86
D. carthusianorum	<0.05	805.95	<0.05	0.46	<0.05	1127.44	<0.05	98807.66
P. lanceolata	<0.05	702.256	<0.05	0.23	<0.05	304.89	<0.05	2419.85
S. ochroleuca	<0.05	536.18	<0.05	0.58	<0.05	549.81	<0.05	41294.77
T. orientalis	<0.05	888.21	<0.05	0.21	<0.05	153.16	<0.05	1110.59

Figure S10 Relationship between plant size and survival for (a) *Bromus erectus* (b) *Dianthus carthusianorum* (c) *Plantago lanceolata* (d) *Scabiosa ochroleuca* (e) *Tragopogon orientalis*. Different treatment combinations are indicated with different shapes and colors.

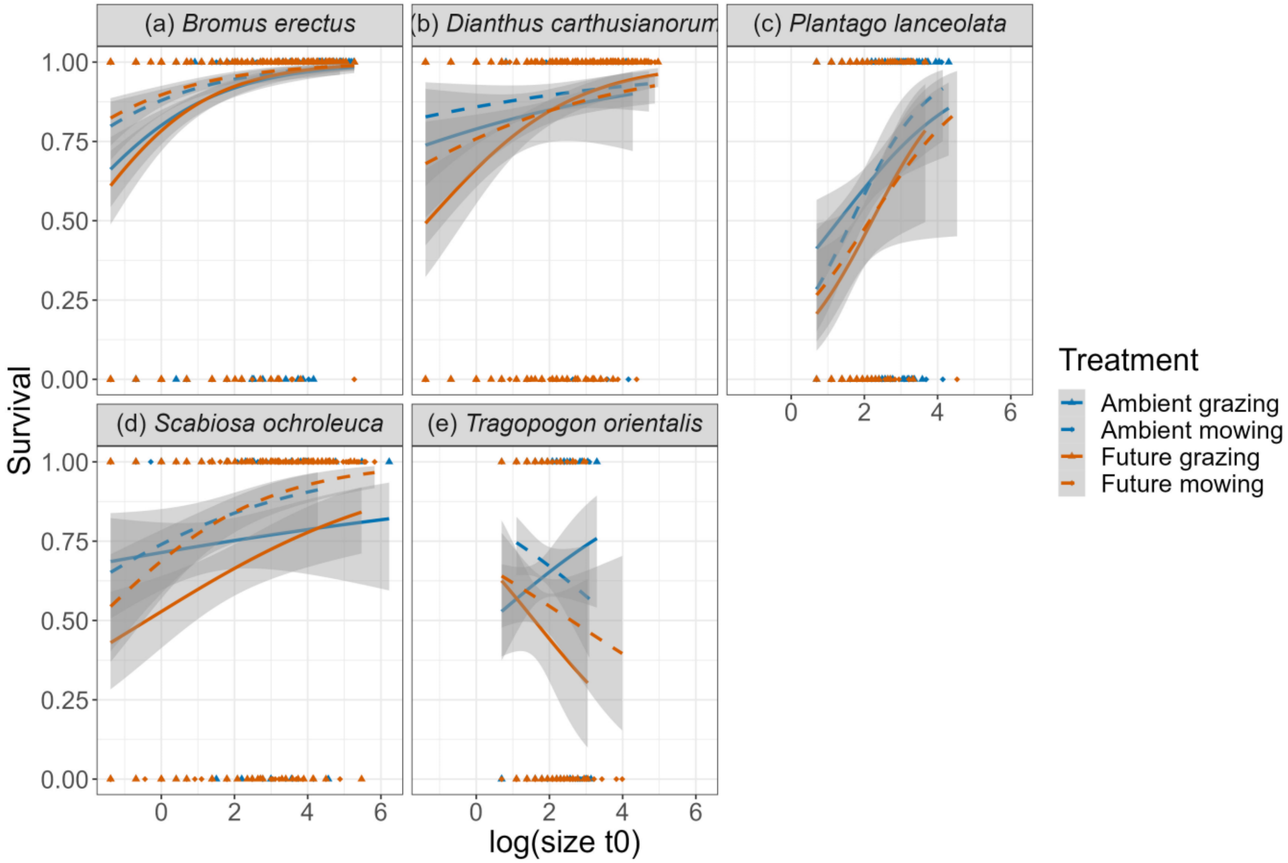

Figure S11 Relationship between plant size in time 0 and plant size in time 1 (i.e., the plant growth vital rates) for (a) *Bromus erectus* (b) *Dianthus carthusianorum* (c) *Plantago lanceolata* (d) *Scabiosa ochroleuca* (e) *Tragopogon orientalis*. Different treatment combinations are indicated with different shapes and colors.

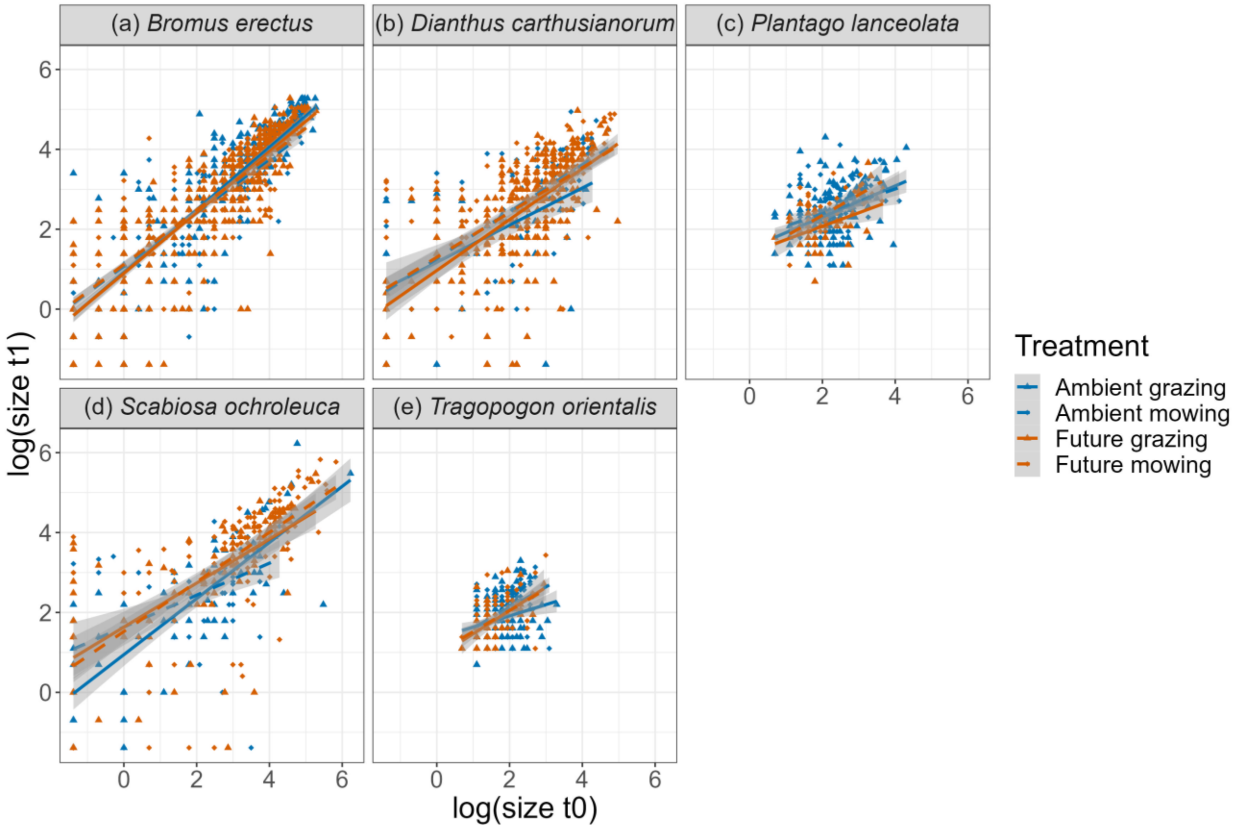

Figure S12 Relationship between plant size and reproduction probability for (a) *Bromus erectus* (b) *Dianthus carthusianorum* (c) *Plantago lanceolata* (d) *Scabiosa ochroleuca* (e) *Tragopogon orientalis*. Different treatment combinations are indicated with different shapes and colors.

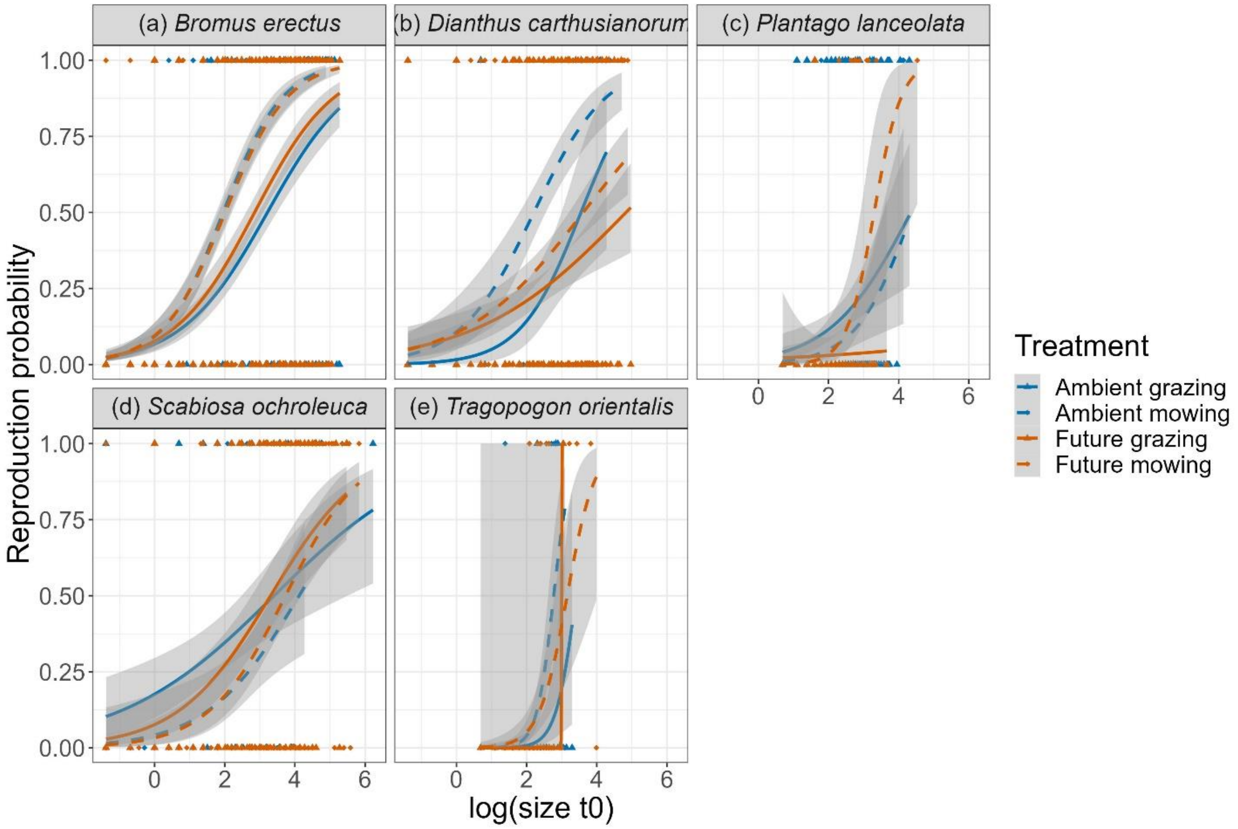

Figure S13 Relationship between plant size and number of seeds for (a) *Bromus erectus* (b) *Dianthus carthusianorum* (c) *Plantago lanceolata* (d) *Scabiosa ochroleuca* (e) *Tragopogon orientalis*. Different treatment combinations are indicated with different shapes and colors.

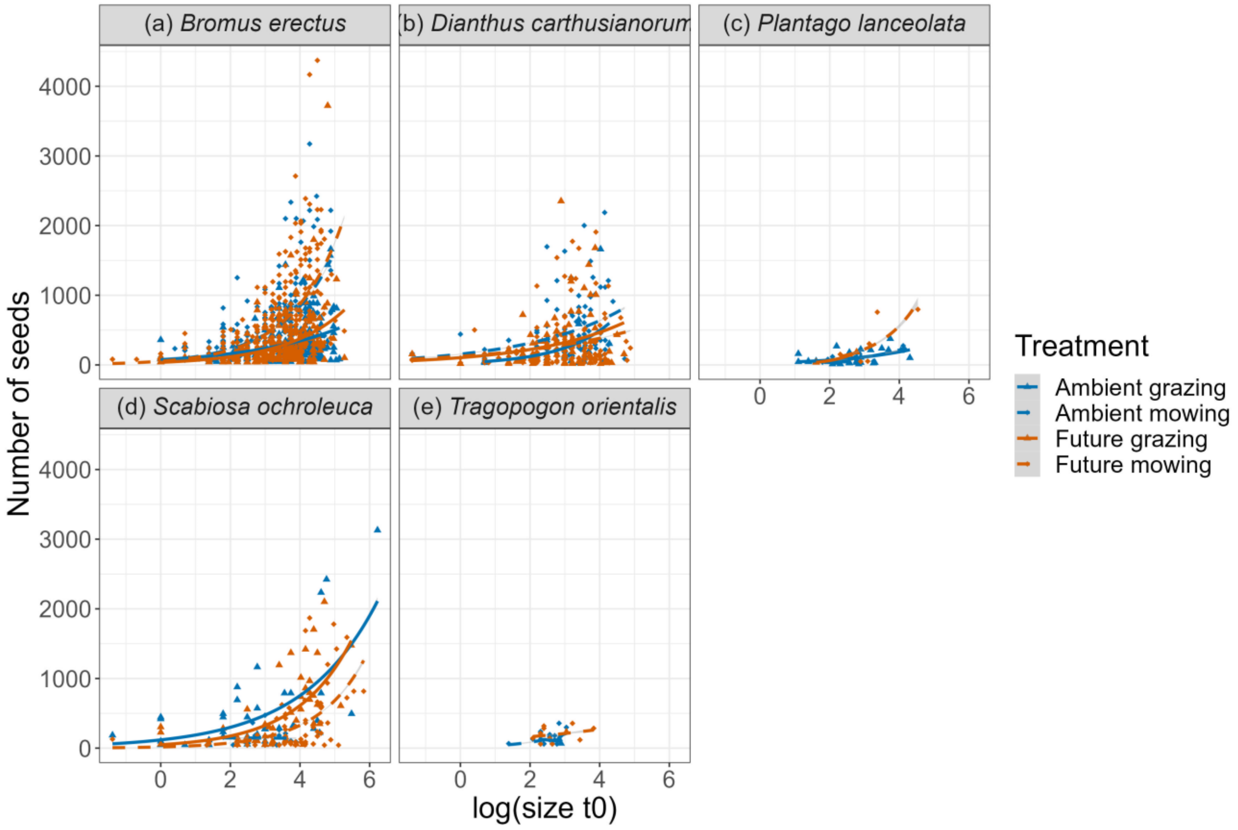

Supplement: Supplementary file 1 — Appendix S1: [file EAP-35-e3063-s001.pdf]
